# Recapitulation of gametic DNA methylation and its post-fertilization maintenance with reassembled DNA elements at the mouse *Igf2/H19* locus

**DOI:** 10.1186/s13072-019-0326-1

**Published:** 2020-01-14

**Authors:** Hitomi Matsuzaki, Daichi Kuramochi, Eiichi Okamura, Katsuhiko Hirakawa, Aki Ushiki, Keiji Tanimoto

**Affiliations:** 1 Faculty of Life and Environmental Sciences, Life Science Center for Survival Dynamics, Tsukuba Advanced Research Alliance (TARA), University of Tsukuba, Tennoudai 1-1-1, Tsukuba, Ibaraki 305-8577 Japan; 2 Graduate School of Life and Environmental Sciences, University of Tsukuba, Tsukuba, Ibaraki Japan; 3 Department of Stem Cells and Human Disease Models, Research Center for Animal Life Science, Shiga University of Medical Science, Otsu, Shiga Japan

## Abstract

**Background:**

Paternal allele-specific DNA methylation of the *H19* imprinting control region (ICR) regulates imprinted expression of the *Igf*2/*H19* genes. The molecular mechanism by which differential methylation of the *H19* ICR is established during gametogenesis and maintained after fertilization, however, is not fully understood. We previously showed that a 2.9-kb *H19* ICR fragment in transgenic mice was differentially methylated only after fertilization, demonstrating that two separable events, gametic and post-fertilization methylation, occur at the *H19* ICR. We then determined that CTCF/Sox-Oct motifs and the 478-bp sequence of the *H19* ICR are essential for maintaining its maternal hypomethylation status and for acquisition of paternal methylation, respectively, during the post-fertilization period.

**Results:**

Using a series of 5′-truncated *H19* ICR transgenes to dissect the 478-bp sequence, we identified a 118-bp region required for post-fertilization methylation activity. Deletion of the sequence from the paternal endogenous *H19* ICR caused loss of methylation after fertilization, indicating that methylation activity of the sequence is required to protect endogenous *H19* ICR from genome-wide reprogramming. We then reconstructed a synthetic DNA fragment in which the CTCF binding sites, Sox-Oct motifs, as well as the 118-bp sequence, were inserted into lambda DNA, and used it to replace the endogenous *H19* ICR. The fragment was methylated during spermatogenesis; moreover, its allele-specific methylation status was faithfully maintained after fertilization, and imprinted expression of the both *Igf2* and *H19* genes was recapitulated.

**Conclusions:**

Our results identified a 118-bp region within the *H19* ICR that is required for de novo DNA methylation of the paternally inherited *H19* ICR during pre-implantation period. A lambda DNA-based artificial fragment that contains the 118-bp sequence, in addition to the previously identified *cis* elements, could fully replace the function of the *H19* ICR in the mouse genome.

## Background

Genomic imprinting, in which a subset of genes is monoallelically expressed in a manner specific to the parent of origin, is a prominent epigenetic phenomenon in mammals. This form of regulation is essential for normal development; hence, its dysregulation causes human diseases, including Beckwith–Wiedemann (BWS) and Silver–Russell syndromes (SRS) [1, 2].

The most common molecular mechanism for achieving genomic imprinting is allele-specific DNA methylation of the imprinting control regions (ICRs), which is frequently observed at imprinted gene loci. DNA methylation of the ICRs is generally acquired during either spermatogenesis or oogenesis; accordingly, ICRs are classified as germline differentially methylated regions (gDMRs). The allelic methylation pattern is maintained after fertilization, throughout the lifespan: the germline-methylated ICRs on one of the alleles are resistant to genome-wide demethylation activity, which is associated with epigenetic reprogramming during the pre-implantation period, and non-methylated ICRs on the other allele are protected from allele-nonspecific de novo methylation during cell differentiation in post-implantation embryos. In other words, differential methylation of the ICRs is regulated at three distinct stages: gametogenesis, pre-implantation, and post-implantation, ensuring monoallelic gene expression in somatic cells.

At the *Igf2/H19* locus, *Igf2* is expressed only from the paternal allele, whereas *H19* is expressed only from the maternal allele [3, 4]. The imprinted expression of both genes is governed by the concerted action of their shared enhancer, located downstream of the *H19* gene, and a paternally methylated gDMR called the *H19* ICR. On the maternal allele, the unmethylated *H19* ICR recruits CCCTC binding factor (CTCF) to form an enhancer-blocking insulator to interfere with distal *Igf2* gene activation by the enhancer, resulting in exclusive *H19* gene expression. By contrast, a hypermethylated paternal ICR silences nearby *H19* gene transcription, but allows *Igf2* gene expression by preventing CTCF from binding to the ICR [5–9]. Loss and gain of methylation at the *H19* ICR has been reported in 30–60% and 5% of patients with SRS and BWS, respectively [2]; therefore, it is of considerable clinical importance to elucidate the allele-specific methylation mechanisms of the *H19* ICR.

In previous work, we generated transgenic mice (TgMs) harboring either randomly integrated mouse *H19* ICR fragments [10] or fragments of the *H19* ICR embedded in human β-globin locus YACs (150 kb; [11]), and found that paternally inherited Tg fragments acquired DNA methylation after fertilization even though they were not methylated in sperm. In other words, our results demonstrated that two separable methylation acquisition processes occurred at the *H19* ICR: one during spermatogenesis that depends on the activity of the surrounding sequence (i.e., outside the *H19* ICR), and another in post-fertilization embryos that is governed by its intrinsic activity. The latter allele-specific, post-fertilization de novo DNA methylation of the transgenic *H19* ICR was also observed in the endogenous *Igf2/H19* gene locus and was catalyzed by oocyte-derived de novo methyltransferases (Dnmt3a and Dnmt3L) [12]. We then determined that a 765-bp sequence in the 5′-portion of the *H19* ICR was necessary for acquisition of methylation after fertilization: deletion of that sequence from the endogenous paternal ICR caused loss of methylation at the remaining *H19* ICR in pre-implantation embryos, without changing its hypermethylation status in sperm. We concluded that paternal allele-specific de novo methylation activity maintains the imprinted methylation of the *H19* ICR in pre-implantation embryos [12]. On the other hand, mutation of CTCF-binding sites [13] and Sox-Oct motifs [14] within the mouse *H19* ICR caused aberrant gain of methylation on the maternal allele after implantation, indicating that these elements are required to protect the maternal, hypomethylated *H19* ICR from allele-nonspecific de novo methylation.

The regulatory sequences we have identified thus far (a 478-bp segment of the 765-bp fragment mentioned above, the CTCF-binding sites and Sox-Oct motifs) in the *H19* ICR are capable of transforming a normally nonimprinted λ DNA sequence into the DMR, when they are assembled together on a λ DNA fragment and assayed in TgM [15]. However, it remains to be determined whether the synthetic fragment can fully reproduce the genomic imprinting phenomena at endogenous mouse *Igf2/H19* gene locus, as allele-specific methylation of the fragment was observed only during the post-fertilization period in the transgenic β-globin gene locus.

In addition, it remains unknown how paternal *H19* ICR methylation is acquired through the 478-bp sequence after fertilization. The sequence could be recognized by a sequence-specific DNA-binding factor(s) in an allele-specific manner, so that the *H19* ICR is distinct from the other genomic regions. Although ZFP57 maintains hypermethylation at multiple ICRs [16, 17], that factor is not a plausible candidate for the regulator of *H19* ICR de novo methylation for two main reasons. First, ZFP57 binds to DNA in a CpG methylation-dependent manner, whereas the transgenic *H19* ICR in sperm is unmethylated. Consistent with this, in our previous work [15], we failed to demonstrate ZFP57 binding to the 478-bp sequence in gel-shift assays. In addition, the DNA methylation status of the endogenous *H19* ICR is not affected in *Zfp57*-knockout mice [16, 18]. Therefore, we assume that currently unidentified factors are responsible for allele-specific, post-fertilization methylation at the *H19* ICR.

In this study, to clarify the mechanisms involved in the two separate mechanisms of gamete DNA methylation and post-fertilization methylation, we generated TgMs carrying a series of 5′-truncated *H19* ICR fragments, with the aim of identifying the *cis* element(s) (and trans-acting factors that bind these elements, ultimately) responsible for the acquisition of post-fertilization methylation. We determined that a 118-bp sequence within the 478-bp region was essential for the activity in the TgM context. As anticipated, deletion of the sequence from the endogenous mouse *H19* ICR decreased its methylation level in pre-implantation embryos, but not in sperm. The λ-based reconstituted fragment, including the 118-bp sequence, recapitulated both imprinted methylation and imprinted gene expression after fertilization in transgenic animals. Most importantly, the reconstituted fragment fully complemented the function of the endogenous *H19* ICR, including acquisition of methylation in sperm.

## Results

### A 118-bp sequence at the 5′-segment of the *H19* ICR is essential for acquisition of paternal methylation

**Fig. 1:**
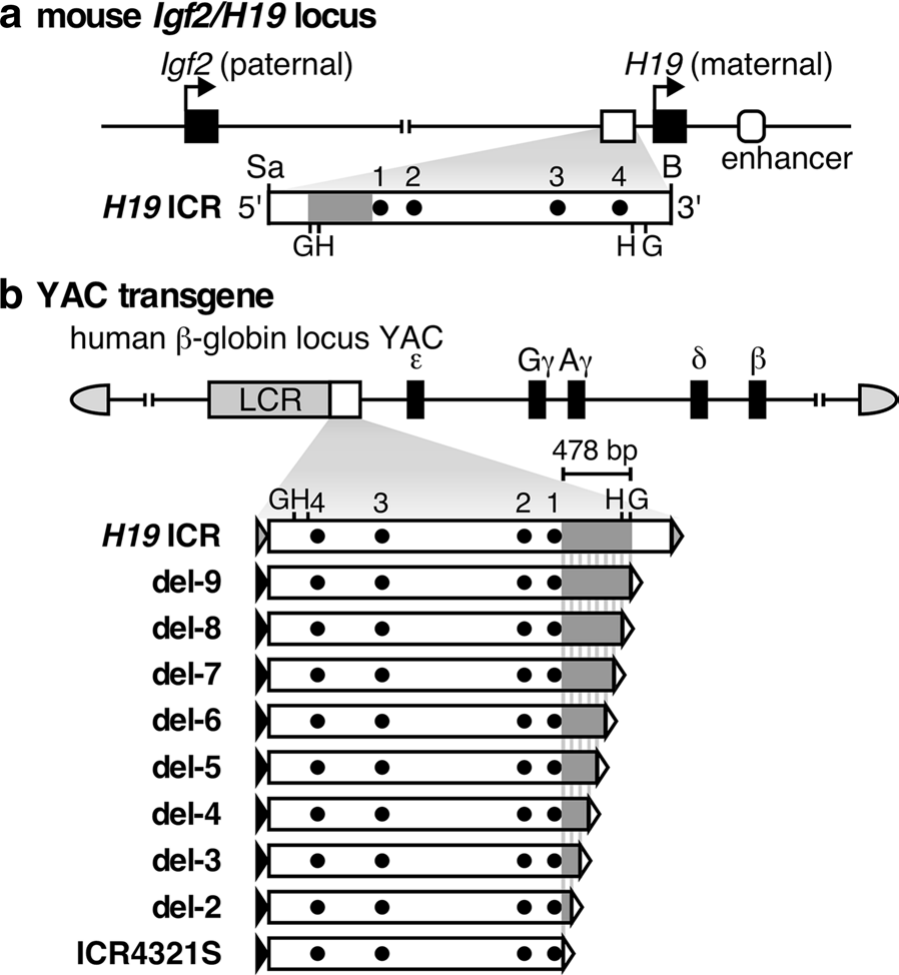
Search for DNA sequences which are responsible for paternal methylation of the *H19* ICR fragment. **a** Structure of the mouse endogenous *Igf2/H19* locus. The expression of paternal *Igf2* and maternal *H19* genes depends on the shared 3′ enhancer. The *H19* ICR, located approximately at − 4 to − 2 kb relative to the transcription start site of *H19* gene is contained within a 2.9-kb SacI (Sa)-BamHI (B) fragment. DNA sequence (478 bp) shown to be sufficient for acquiring paternal methylation in TgM [15] is marked in gray. Dots (1–4) indicate CTCF-binding sites. G, BglII; H, HindIII sites. **b** Structure of the 150-kb human β-globin locus YAC. The LCR and β-like globin genes are denoted as gray and filled boxes, respectively. In our previous studies, the *H19* ICR (2.9-kb) or the ICR4321S (766-bp shorter than the original 2.9-kb sequence) fragments were introduced 3′ to the LCR [11, 12]. In this study, a series of 5′-truncated *H19* ICR fragments (del-2–9) were inserted into the identical position of the YAC to examine their activities in TgM

In previous work, we have narrowed down the sequence responsible for post-fertilization paternal methylation of the *H19* ICR to a 478-bp region and demonstrated that it is required in vivo for normal development (Fig. 1a; [12, 15]). Furthermore, we showed that post-fertilization, allele-specific methylation was recapitulated in a λ-phage-based synthetic DNA fragment in the TgM only when the 478-bp sequence was included [15]. To further define the responsible sequence in this study, we generated *H19* ICR fragments with a series of 5′-deletions of the 478-bp region (~ 60 bp intervals) and inserted them into the human β-globin YAC to generate TgMs (Fig. 1b). To avoid position-of-integration site effects, which transgene fragments frequently incur, and to directly compare activity at a single genomic site, we combined two successive deletion fragments side-by-side and employed a transgene co-placement strategy [19] (Additional file 1: Fig. S1A). Four YAC constructs, each carrying a distinct set of deletions (fragments del-8/9 to 2/3), were used to generate TgMs, and at least two independent mouse lines were established for each construct. Long-range analysis of thymus genomic DNA demonstrated that all but two harbored an intact, single-copy transgene (Additional file 1: Fig. S1B) (lines 36 and 20 of the del-2/3 TgMs lacked sequence 5′ to the LCR and 3′ to the β-globin gene regions, respectively). Cross-mating of these TgMs with Cre-TgM caused in utero Cre-loxP recombination that generated daughter sublines carrying either of the *H19* ICR deletion fragments, which was confirmed by Southern blot analysis of somatic cell DNA (Additional file 1: Fig. S1C and D).

Tail somatic cell DNA of animals inheriting the YAC transgenes either paternally or maternally was prepared, and the methylation status of their endogenous and transgenic *H19* ICR sequences was determined by Southern blot analysis (Additional file 2: Fig. S2A). The appearance of digested and undigested endogenous fragments in equimolar ratios served as a control for complete genomic DNA digestion by methylation-sensitive restriction enzymes. The results revealed that 5′-deletion fragments of the *H19* ICR in animals inheriting the transgenes maternally were hypomethylated, as was the intact 2.9-kb fragment (mat. in Additional file 2: Fig. S2B–F). By contrast, while the paternally inherited transgenic *H19* ICR in the del-9 and del-8 lines was hypermethylated (pat. in Additional file 2: Fig. S2B and C), those in the del-7 lines (pat. in Additional file 2: Fig. S2D) exhibited partial methylation, and all others were hypomethylated (pat. in Additional file 2: Fig. S2E–I).

**Fig. 2:**
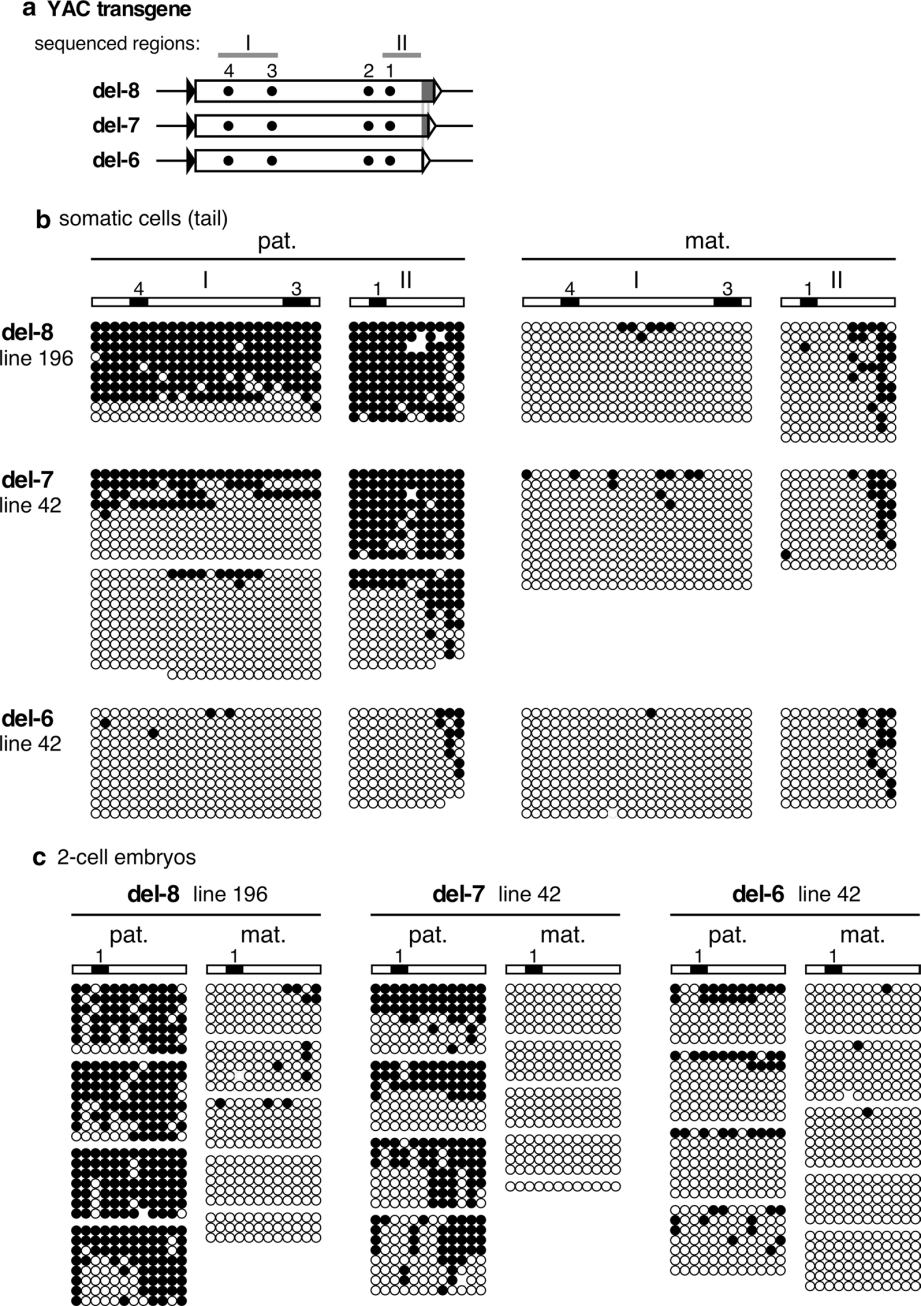
DNA methylation status of the del-8, 7, and 6 fragments in YAC TgM. **a** Map of the transgene fragments. Region differences (118 bp) among three constructs are shown in gray. Regions I and II that were analyzed by bisulfite sequencing in **b** and **c** were indicated by gray bars above the map. **b** Tail genomic DNAs of TgM inheriting the transgenes either paternally (pat.) or maternally (mat.) were pooled according to the transgene’s parental origin and subjected to bisulfite sequencing, the methylation levels of which were determined beforehand by Southern blotting in Additional file 2: Fig. S2. Each horizontal row represents a single DNA template molecule. Methylated and unmethylated CpG motifs are shown as filled and open circles, respectively. Position of CTCF-binding sites is shown by filled boxes. **c** Two-cell embryos that inherited the transgenes either paternally (pat.) or maternally (mat.) were embedded in agarose beads and treated with sodium bisulfite. The beads were used to amplify the region II in **a** by nested PCR. PCR products were individually subcloned and sequenced. The results from single beads are presented together in a cluster

To precisely determine the sequence requirements for paternal *H19* ICR methylation, we analyzed the methylation status of the Tg *H19* ICR in lines del-8, -7, and -6 by bisulfite sequencing (Fig. 2a). Analysis of tail somatic cell DNA revealed that paternally inherited Tg sequences in lines del-8, del-7, and del-6 were hyper-, partially and hypo-methylated, respectively, whereas all of these sequences were hypomethylated when maternally inherited (Fig. 2b). To determine whether loss of methylation in the paternal del-7 and del-6 sequences was due to a lack of de novo DNA methylation activity in pre-implantation embryos or a lack of methylation maintenance activity after implantation, we analyzed two-cell embryos (Fig. 2c). The del-7 and del-6 sequences were partially hypomethylated, suggesting that the 118-bp sequence between the 5′ ends of the del-8 and del-6 fragments are essential for de novo methylation of the paternal *H19* ICR during the pre-implantation period.

### Deletion of the 118-bp sequence results in loss of methylation at the *H19* ICR during the pre-implantation period

**Fig. 3:**
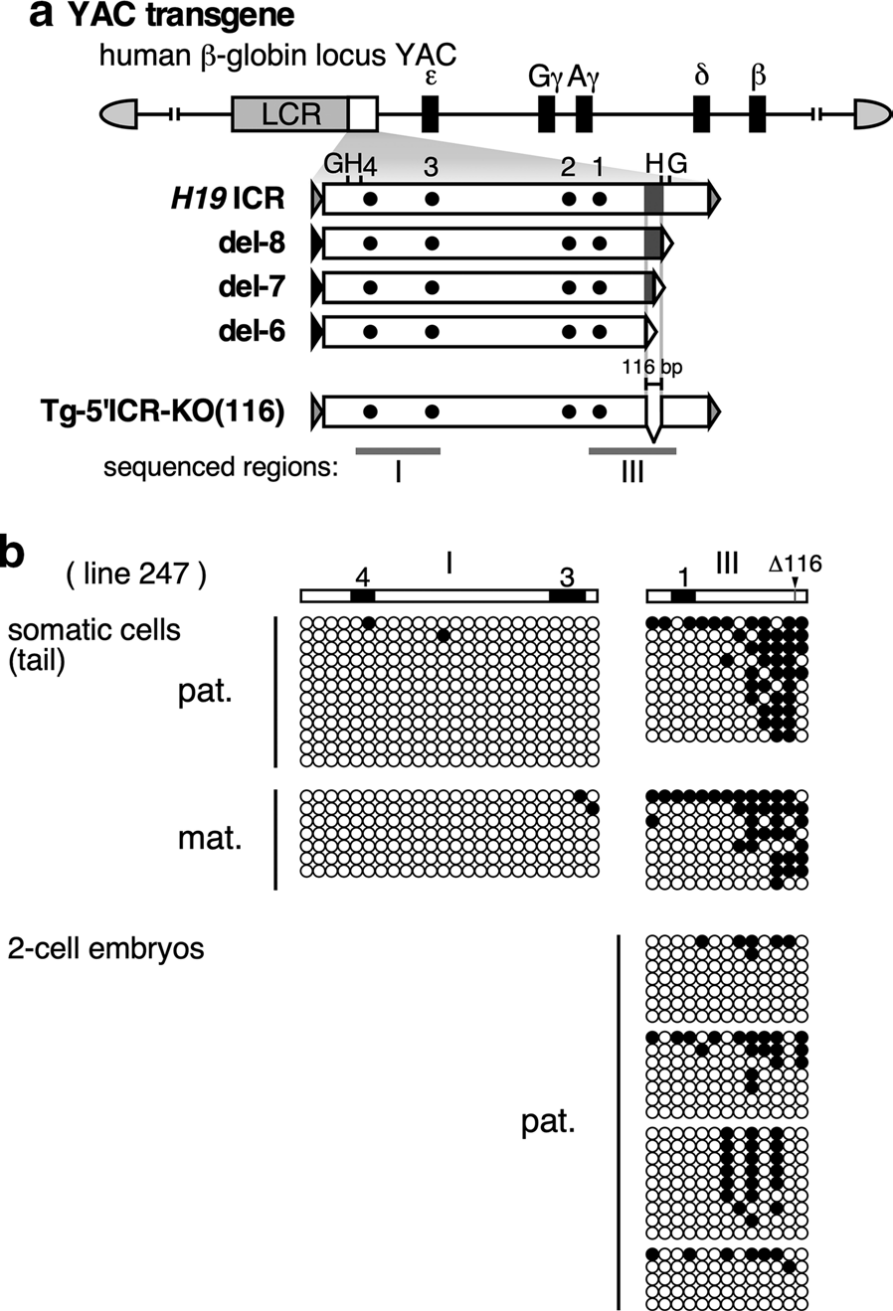
The 118-bp sequence was required for paternal methylation of the transgenic *H19* ICR. **a** Structure of the transgenes. The 116-bp sequence, which was a part of the 118-bp region identified by 5′-truncation experiments (Fig. 2), was internally deleted from the 2.9-kb *H19* ICR fragment in TgM by CRISPR/Cas9 genome editing (Tg-5′ICR-KO(116)). Regions I and III, indicated by gray bars below the map, were analyzed by bisulfite sequencing. **b** DNA methylation status of the mutant *H19* ICR transgene in tail somatic cells (upper panel) or 2-cell embryos (lower) of TgM, that inherited the transgenes either paternally (pat.) or maternally (mat.), was analyzed by bisulfite sequencing

To confirm our results in the 5′-deletion mutants, we next deleted the sequence from the 2.9-kb *H19* ICR in YAC TgM by in vivo genome editing (Fig. 3a). To this end, we generated two pX330-based plasmids expressing the Cas9 nuclease and guide RNAs targeting the 5′ ends of the del-6 and del-8 sequences (Additional file 3: Fig. S3A). Pronuclear co-injection of these plasmids into fertilized eggs recovered from the *H19* ICR/human β-globin YAC TgM [11] led to generation of mutant transgenic loci. Two mutant lines with an identical 116-bp deletion (a bit shorter than 118 bp due to restriction by PAM motif locations) in the *H19* ICR were generated (lines 226 and 247; Additional file 3: Fig. S3A). Analysis of tail somatic cell DNA by Southern blotting and bisulfite sequencing revealed that the mutant transgenic *H19* ICR sequences were hypomethylated regardless of their parental origin (Additional file 3: Fig. S3B and C, and Fig. 3b). In addition, hypomethylation of the mutant paternal *H19* ICR was also observed in two-cell embryos (Fig. 3b), indicating that the 116-bp sequence was required for post-fertilization methylation of the *H19* ICR transgene.

**Fig. 4:**
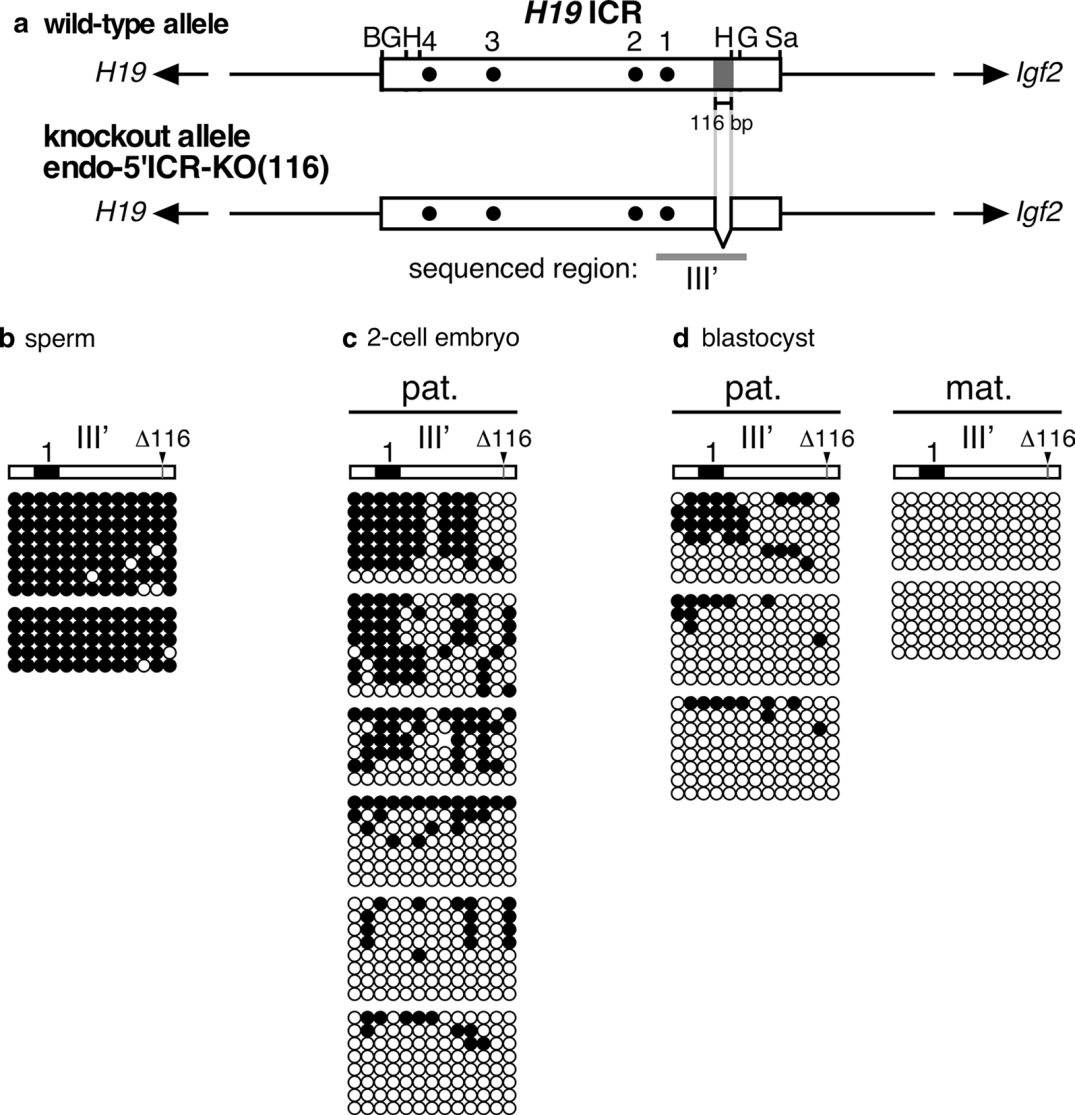
The 118-bp sequence was also required for paternal methylation of the endogenous *H19* ICR. **a** Map of wild-type and knockout alleles. The same 116-bp sequence that was deleted from the transgenic *H19* ICR fragment in Fig. 3, was removed from the mouse endogenous locus by CRISPR/Cas9 genome editing (endo-5′ICR-KO(116)). **b**–**d** DNA methylation status of the mutant *H19* ICR in sperm (**b**), 2-cell embryos (**c**), or blastocysts (**d**) of 116-bp KO mice, that inherited the KO allele either paternally (pat.) or maternally (mat.), was analyzed by bisulfite sequencing

When we generated the aforementioned mutation in the transgenic *H19* ICR, endogenous *H19* ICR was concomitantly mutagenized, and the 116-bp sequence was deleted (Fig. 4a). Bisulfite sequencing analysis revealed that the endogenous *H19* ICR with the mutation was fully methylated in sperm (Fig. 4b), demonstrating that the 116-bp sequence was dispensable for its germline methylation. This result was consistent with our hypothesis that establishment of methylation at the 2.9-kb *H19* ICR sequence in sperm was under the control of its surrounding sequence, rather than its intrinsic activity [10]. By contrast, paternally inherited mutant *H19* ICR exhibited significant loss of methylation in two-cell (Fig. 4c) and blastocyst stage embryos (Fig. 4d). This result was also consistent with our hypothesis that the post-fertilization methylation activity of the *H19* ICR observed in the TgM context was responsible for protecting its germline-established paternal methylation (at the endogenous locus) against genome-wide reprogramming activity during pre-implantation period [12]. In addition, these results clearly demonstrated that the paternal *H19* ICR methylation cannot be maintained solely by a ZFP57-mediated mechanism during the post-fertilization period, as the mutant sequence retained all binding sites for ZFP57 [15].

### The reconstituted synthetic fragment recapitulates genomic imprinting in YAC-TgM

We previously showed that artificial DMR activity can be generated by assembling the sequences required for protecting the paternal *H19* ICR against genome-wide demethylation (by simultaneous de novo DNA methylation) during the pre-implantation period (i.e., the 478-bp sequence), as well as those required for protecting the maternal, unmethylated *H19* ICR from post-implantation de novo methylation (i.e, the CTCF and Sox-Oct motifs) on the λ DNA sequence [15]. To determine whether the shorter 118-bp sequence was sufficient to confer the same activity, we combined the LCb fragment (λ DNA fragment harboring the CTCF and Sox-Oct motifs; [14, 15]) and the LCb fragment with the 118-bp sequence attached (termed the LCb118), and inserted them into human β-globin YAC, employing the transgene co-placement strategy to precisely compare their activities (Additional file 4: Fig. S4A). Following establishment of two intact, single-copy YAC TgM lines, confirmed by long-range Southern blot analysis of thymic DNAs (lines 28 and 890; Additional file 4: Fig. S4B), the mice were crossed with Cre-TgM to induce in utero Cre-loxP recombination. Tail DNAs from the offspring confirmed that Tg sublines harboring either LCb or LCb118 sequences were successfully obtained from both parental lines (Additional file 4: Fig. S4C).

Methylation analysis of tail somatic cell DNA by Southern blotting revealed that the LCb fragments exhibited low-level methylation in more than half of the individuals inheriting the transgene paternally (lines 28 and 890; Additional file 5: Fig. S5A and B), consistent with previous data obtained at distinct integration sites of transgenes [15]. By contrast, the paternally inherited LCb118 fragments exhibited high-level methylation in all individuals analyzed (lines 28 and 890; Additional file 5: Fig. S5C and D), whereas maternally inherited fragments exhibited hypomethylation, which was also the case for the LCb (Additional file 5: Fig. S5B). Importantly, the methylation status of LCb118 transgenes was reprogrammable over generations depending on parental origin, which is an important feature of genomic imprinting (Additional file 5: Fig. S5D). Therefore, we concluded that the 118-bp sequence was sufficient for the acquisition of paternal methylation. In addition, LCb and LCb118 fragments were not methylated in the testis germ cells (Additional file 6: Fig. S6), indicating that the 118-bp sequence conferred post-fertilization acquisition of methylation.

**Fig. 5:**
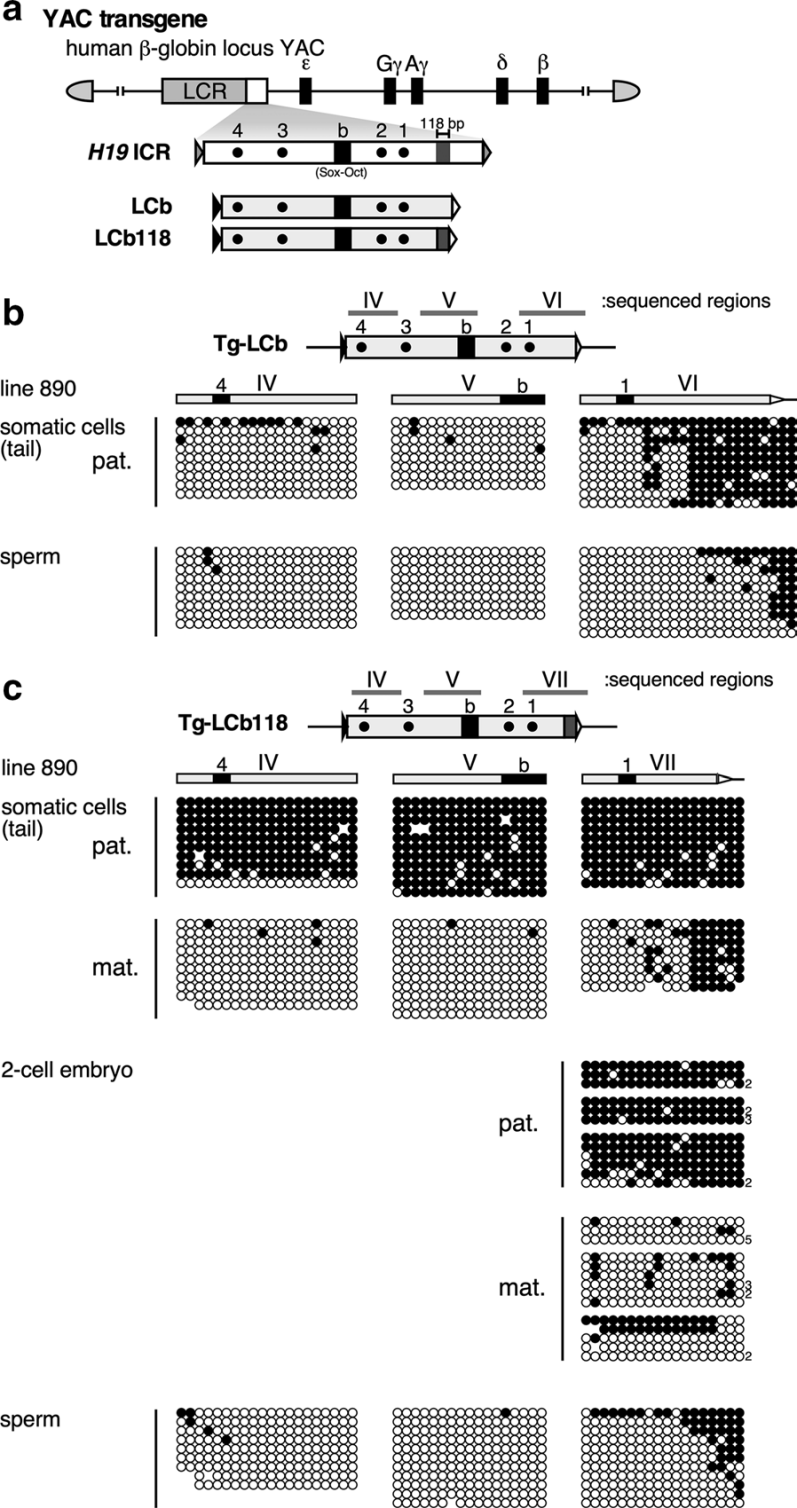
Reconstitution of the differentially methylated region by a synthetic DNA fragment in TgM. **a** TgM lines were generated by using the 150-kb human β-globin locus YAC bearing either the LCb or LCb118 fragments introduced 3′ to the LCR within the YAC. Filled and gray boxes indicate the “b” region (which includes Sox-Oct motifs) and 118-bp sequence, respectively. **b**, **c** DNA methylation statuses of the LCb (**b**) and LCb118 (**c**) fragments in tail somatic cells, 2-cell embryos, and sperm of YAC-TgM, that inherited the transgenes either paternally (pat.) or maternally (mat.). Regions analyzed by bisulfite sequencing were indicated by gray bars above each map

To precisely evaluate the function of the 118-bp sequence in the context of artificially assembled LCb sequences embedded in the YAC Tg in mice (Fig. 5a), we analyzed their methylation statuses by bisulfite sequencing. The results revealed that the Tg-LCb sequence was not methylated in the paternal allele in tail somatic cells and in the sperm (Fig. 5b). By contrast, the Tg-LCb118 sequence in tail somatic cells was hypermethylated only after paternal transmission (Fig. 5c). In addition, such acquisition of allele-specific methylation was observed as early as the two-cell stage, whereas the sequence was not methylated in sperm (Fig. 5c). Thus, given that the temporal and allele specificity of methylation acquisition at the transgenic *H19* ICR and transgenic LCb118 sequences were identical, we concluded that paternal allele-specific post-fertilization methylation was recapitulated by the synthetic LCb118 sequence in the TgM context.

**Fig. 6:**
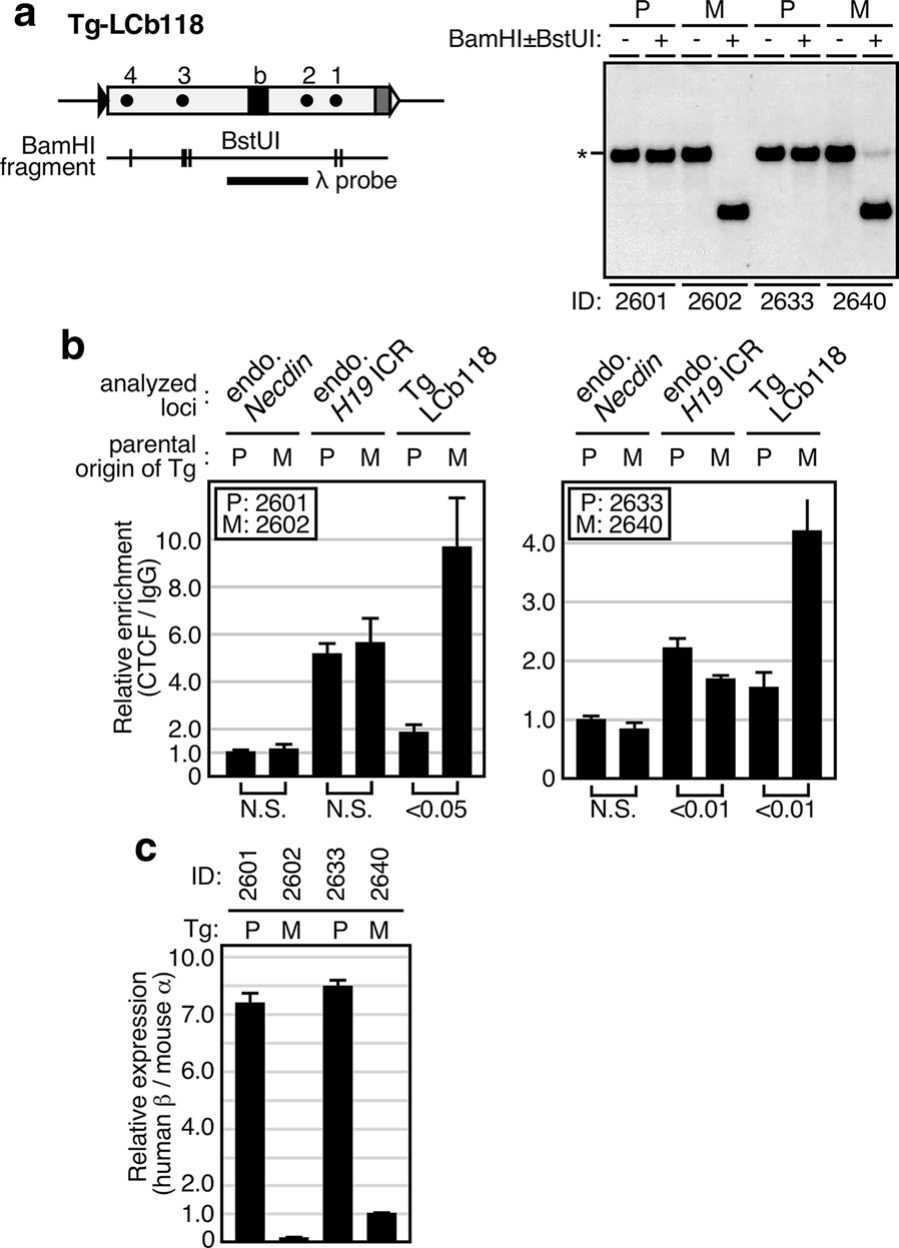
Genomic imprinting recapitulated in the LCb118 YAC-TgM. **a**–**c** Two pairs of LCb118-TgM (ID No. 2601/2602 and 2633/2640), each inheriting the transgene either paternally (P) or maternally (M) were made anaemic and spleens were removed, from which one-quarter each was used for genomic DNA or total RNA preparation with the remaining half used for chromatin preparation. **a** DNA methylation status of the transgene was determined by Southern blot analysis using BamHI with (+) or without (−) BstUI (vertical lines) and a *λ* probe. *: methylated, uncut fragments in BstUI (+) lanes. **b** ChIP analysis of CTCF occupancy at the transgene. Chromatin was immunoprecipitated using either control IgG or anti-CTCF antibodies. Following qPCR analyses of three distinct genomic regions (*Necdin*; negative control, endogenous *H19* ICR; positive control, and LCb118 transgene), relative enrichment values (CTCF/IgG signal ratio) were calculated. The average and standard deviation (S.D.), determined by three reactions, are depicted, as a signal for *Necdin* (M) was arbitrary set at 1.0. Statistical differences were determined using an unpaired *t* test (N.S., not significant). **c** The relative expression levels of the human β-globin gene, after normalization to that of the endogenous mouse α-globin gene were determined by RT-qPCR analysis. The average and standard deviation (S.D.), determined by three reactions, are depicted, as a value of No. 2640 animal was arbitrary set at 1.0

The LCb118 sequence was placed between the LCR enhancer and the genes in the human β-globin locus YAC in TgM. Therefore, we anticipated monoallelic β-globin gene expression because of allele-specific enhancer-blocking activity due to methylation-sensitive CTCF binding to LCb118. To test the in vivo role of LCb118, we prepared chromatin from nucleated erythroid cells of adult animals inheriting the transgene either paternally or maternally; their hyper- and hypomethylation states, respectively, were confirmed by Southern blot analysis (Fig. 6a). ChIP analysis of the chromatin revealed that CTCF binding was enriched at a significantly higher level at the maternal LCb118 than at the paternal one (Fig. 6b), consistent with the respective methylation statuses of the sequences. Level of enrichment at the endogenous *H19* ICR was similar regardless of whether the transgene was inherited paternally or maternally; this is as expected, as the value represents the sum of the two parental alleles. We then analyzed transgenic human β-globin gene expression in the nucleated erythroid cells by RT-qPCR. The results revealed that the transgene was highly expressed only when paternally transmitted (Fig. 6c).

### The reconstituted fragment is able to replace the function of the endogenous *H19* ICR

We previously demonstrated that the post-fertilization de novo methylation activity of the *H19* ICR protects its paternal methylation status against pre-implantation reprogramming of the whole genome. However, in the TgM context, acquisition of methylation in sperm took place neither in the wild-type nor in artificially assembled fragments. Based on our previous observations [10], we anticipated that gametic methylation of the *H19* ICR was under control of the surrounding sequences somewhere within the *Igf2/H19* gene locus. Therefore, we decided to test whether the LCb/LCb118 sequences could be methylated in sperm when they were inserted in place of the endogenous *H19* ICR sequence, and if so, whether they could completely replace its function (Additional file 7: Fig. S7A). To generate knock-in alleles, LCb or LCb118 targeting vectors harboring the *H19* ICR flanking sequences, together with a genome editing plasmid targeting the *H19* ICR region, were transfected into C57BL/6 (B6) mouse ES cells. Southern blot and sequencing analyses identified one and two ES cell clones, respectively, with their endogenous *H19* ICR sequences correctly replaced with the LCb or LCb118 sequences (Additional file 7: Fig. S7B and data not shown). These ES cell clones were then used for co-culture aggregation to establish mouse lines. Correctness of mutagenesis was confirmed by Southern blot and sequencing analyses of the mouse tail tip DNA (Additional file 7: Fig. S7C and data not shown).

**Fig. 7:**
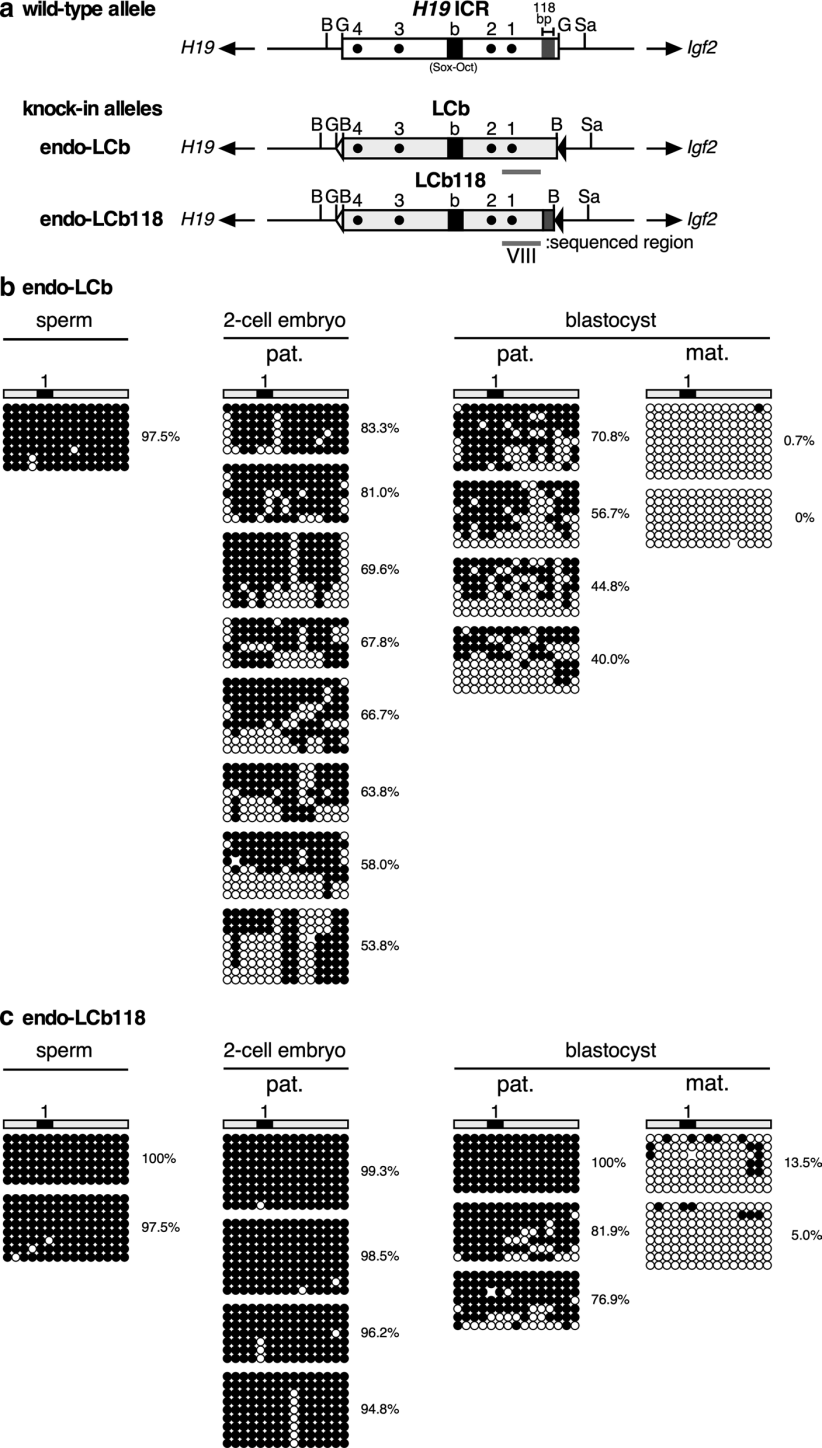
Reconstitution of the differential methylation in the LCb118 sequence at the mouse endogenous *Igf2/H19* locus. **a** Map of the wild-type and knock-in alleles. The endogenous mouse *H19* ICR sequence was replaced by the LCb or LCb118 synthetic DNA fragments. Filled and gray boxes indicate the “b” region (which includes Sox-Oct motifs) and 118-bp sequence, respectively. DNA methylation status of the LCb (**b**) and LCb118 (**c**) sequence in sperm, 2-cell embryos, and blastocysts of knock-in mice, that inherited the mutant alleles either paternally (pat.) or maternally (mat.), was analyzed by bisulfite sequencing. The overall percentage of methylated CpGs is indicated next to each panel

Next, the methylation status of the synthetic sequences knocked in at the endogenous *Igf2/H19* locus was analyzed by bisulfite sequencing (Fig. 7a). As anticipated, the LCb sequence was almost fully methylated in sperm (Fig. 7b), in contrast to its unmethylated state in the transgenic environment (Fig. 5b). After fertilization, however, the methylation level of the paternal LCb sequence gradually decreased during the pre-implantation period (Fig. 7b), consistent with a lack of post-fertilization methylation activity of the LCb (Fig. 5b). The maternally inherited fragment was almost devoid of methylation at the blastocyst stage (Fig. 7b). By contrast, the LCb118 sequence, which was also methylated in the sperm, remained methylated even after fertilization (Fig. 7c). This hypermethylation was also observed at the blastocyst stage, when the methylation level of the maternally inherited sequence was significantly lower (Fig. 7c). Taken together, these observations suggest that at the endogenous *Igf2/H19* locus, hypermethylation of the paternal *H19* ICR established in the sperm was maintained even after fertilization in a 118-bp sequence–dependent manner.

**Fig. 8:**
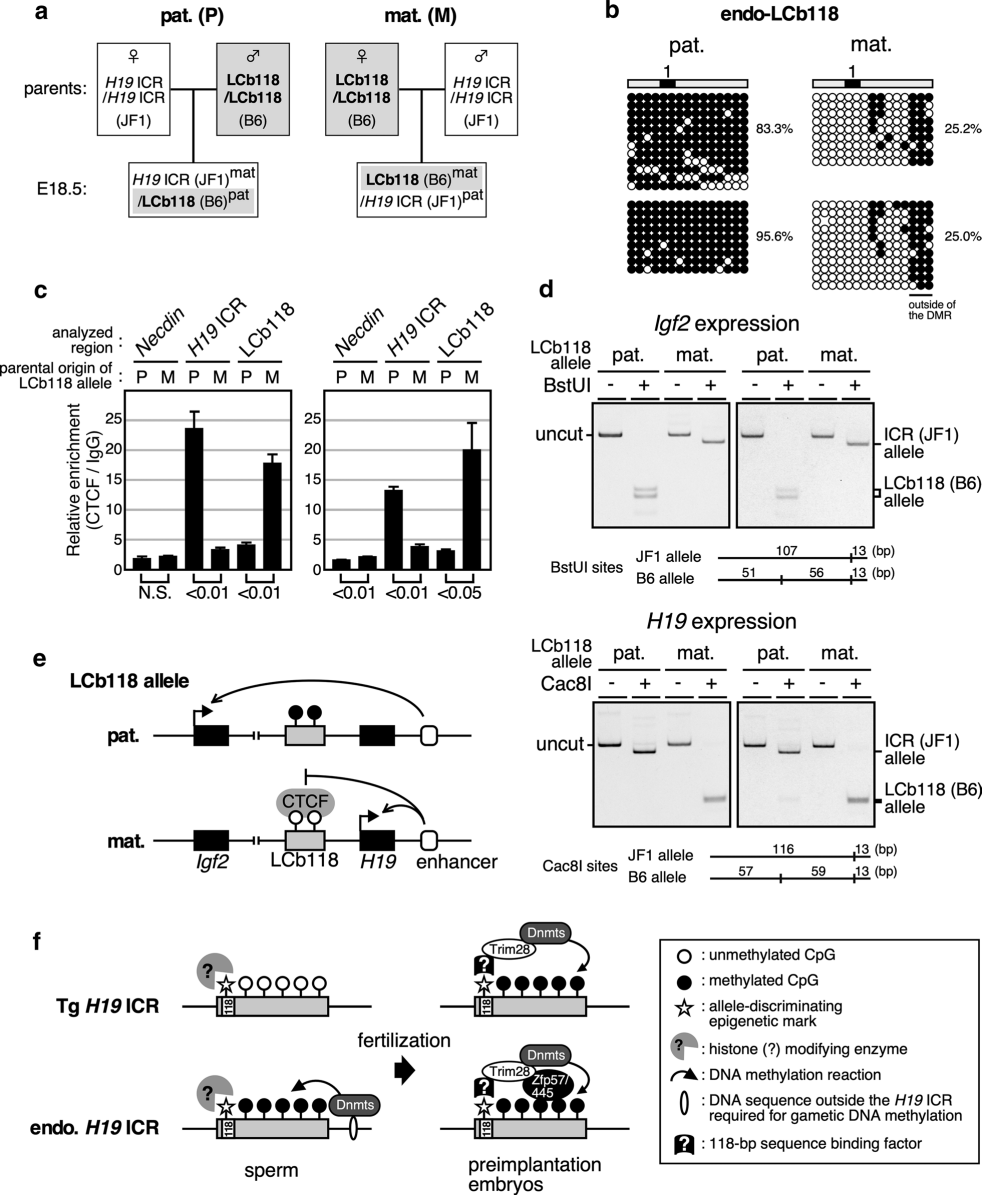
Genomic imprinting recapitulated in LCb118 knock-in mice. **a** Breeding scheme. In order to distinguish parental origin of the alleles by using SNPs between inbred mouse strains, endo-LCb118 homo-knock-in mice (LCb118/LCb118; C57BL/6 J [B6] background) were mated with wild-type mice (*H19* ICR/*H19* ICR; JF1/Msf [JF1]), and offspring was obtained. **b**–**d** Livers from two pairs of E18.5 embryos, each inheriting the knock-in allele either paternally (pat.; P) or maternally (mat.; M) were used for genomic DNA, total RNA, and chromatin preparations, as in Fig. 6. **b** DNA methylation status of LCb118 region (the same position as in Fig. 7a) was analyzed by bisulfite sequencing. **c** ChIP analysis of CTCF occupancy at the LCb118 sequence. Chromatin was immunoprecipitated using either control IgG or anti-CTCF antibodies. Following qPCR analyses of three distinct genomic regions (*Necdin*; negative control, endogenous *H19* ICR; positive control, and LCb118), relative enrichment values (CTCF/IgG signal ratio) were calculated. The average and standard deviation (S.D.), determined by three reactions, are depicted. Statistical differences were determined using an unpaired *t* test (N.S., not significant). **d** The allele-specific expression of the *Igf2* and *H19* genes was examined by RFLP analysis. *Igf2* and *H19* gene transcripts were amplified by RT-PCR followed by BstUI or Cac8I digestions, respectively. Parental origin of transcripts was discriminated by allele-specific restriction sites. The sites were also introduced into primer sequence so that complete digestion of PCR products can be concomitantly monitored. **e** Schematic representation of the genomic imprinting recapitulated in the LCb118 knock-in allele. **f** Hypothetical model for post-fertilization methylation maintenance mechanism at the *Igf2/H19* locus

We then tested whether monoallelic expression (i.e., genomic imprinting) of the *Igf2/H19* genes was recapitulated at the mutant locus. To discriminate allelic expression of the *Igf2* and *H19* genes, we took advantage of single-nucleotide polymorphisms (SNPs) between the B6 and JF1 inbred mouse strains. Male mice homozygous for the LCb118 allele, which was generated in the B6 background, were mated with wild-type JF1 female mice to derive a paternally inherited LCb118 allele in the offspring (Fig. 8a, left), whereas B6 female mice homozygous for the LCb118 allele were mated with wild-type JF1 male mice to derive a maternally inherited LCb118 allele in the offspring (Fig. 8a, right). Bisulfite sequencing analysis of fetal liver DNA (18.5 dpc) revealed that paternally inherited LCb118 was preferentially methylated (Fig. 8b; the methylated region common to both parental transmissions is outside of the DMR).

Next, we conducted a ChIP assay to analyze CTCF binding in the fetal liver (Fig. 8c, 18.5 dpc). When the LCb118 allele was maternally inherited, CTCF bound at significant levels to its sequence. By contrast, when the LCb118 allele was paternally inherited, CTCF was enriched at the maternally inherited WT *H19* ICR. These results clearly demonstrate that CTCF bound the maternally inherited, hypomethylated sequences irrespective of whether they were *H19* ICR or LCb118 (Fig. 8c).

Finally, we analyzed expression of the *Igf2* and *H19* genes by PCR amplification of cDNA prepared from the fetal liver RNA, followed by restriction enzyme digestion at sites containing strain-specific SNPs (Fig. 8d). The results revealed that *Igf2* was expressed only from the alleles carrying either hypermethylated *H19* ICR or LCb118 sequences *in cis* (Fig. 8d, upper), whereas *H19* was active only when *cis*-linked *H19* ICR or LCb118 sequences were hypomethylated (Fig. 8d, bottom). Similar results were obtained when liver RNA from E12.5 embryos was used (Additional file 8: Fig. S8A). By contrast, the *H19* gene was aberrantly expressed from the paternally inherited LCb allele in multiple embryos (Additional file 8: Fig. S8B), in which LCb sequences exhibited lower methylation levels (Additional file 9: Fig. S9). In addition, the number of pups that inherited the LCb allele paternally was significantly smaller than expected (Additional file 10: Table S1). These results suggested that the LCb was insufficient to replace *H19* ICR function in the regulation of genomic imprinting and proper development.

In summary, an artificially reconstituted LCb118 sequence knocked into the endogenous *Igf2/H19* locus faithfully recapitulated the phenomenon of genomic imprinting, including establishment of methylation in the sperm, post-fertilization and post-implantation maintenance of differential methylation, allele-restricted CTCF binding, and control of monoallelic gene expression (Fig. 8e). In addition, our results clarify the role of the 118-bp sequence in the post-fertilization maintenance of paternally inherited endogenous *H19* ICR.

## Discussion

Allele-specific DNA methylation of ICRs plays a fundamental role in the regulation of genomic imprinting. Since most ICRs are differentially methylated during gametogenesis (i.e., gDMR), a great deal of attention has been focused on elucidating the molecular mechanism by which ICRs in primordial germ cells, where almost all pre-existing methylation is erased, eventually acquire asymmetric methylation during germ cell differentiation. Subsequently, genome-wide DNA methylation analysis identified many gDMRs that are methylated by the same mechanism as ICRs, but are unrelated to genomic imprinting [20, 21]. Therefore, a critical difference between general gDMRs and ICRs is whether their differential methylation statuses are maintained after fertilization. In other words, the mechanism that selectively maintains post-fertilization methylation at ICRs defines genomic imprinted regions.

Sequence-dependent DNA-binding proteins are the most plausible candidates to support post-fertilization methylation maintenance at the specified ICRs. In fact, deficiency of ZFP57, one of the KRAB-zinc finger proteins (KZFPs), causes loss of methylation at multiple ICRs [16]. Through binding to its consensus DNA motif (5′-TGCCGC-3′) in the ICRs, ZFP57 maintains methylation via recruitment of a heterochromatic complex that contains KAP1, DNA methyltransferases, and histone methyltransferases [16, 17, 22]. ZFP57 binding to DNA depends on CpG methylation of the consensus motif, which in turn allows the maintenance of DNA methylation in an allele-specific manner [17]. Differential DNA methylation status was not affected at some ICRs, including the *H19* ICR, in *Zfp57*-null mice [16, 18], although ZFP57 binds these ICRs in ES cells [17], suggesting the existence of additional regulatory factors. Most recently, another KZFP, ZFP445, was reported to bind to methylated ICRs and participate in maintenance of their imprinted methylation status [23]. Allele-specific methylation at almost all ICRs was severely affected in *Zfp57*/*Zfp445* double knockout mice, suggesting that these two proteins may coordinately maintain differential DNA methylation.

Despite the existence of recognition motifs for ZFP57, the LCb sequence and the *H19* ICR sequence with a 116-bp deletion partly lost their DNA methylation at the paternal endogenous *Igf2/H19* locus during the post-fertilization period. Therefore, we propose additional mechanism(s) for maintenance of imprinted methylation. In our TgMs with the *H19* ICR fragment, paternal allele–specific DNA methylation occurs at the transgene soon after fertilization [11, 12], indicating that “de novo” methylation takes place in an allele-specific manner at the transgenic *H19* ICR. Consistent with this, the paternally inherited *H19* ICR fails to acquire methylation in early embryos when the supply of de novo methyltransferases, Dnmt3a and Dnmt3L, was eliminated by deletion of the corresponding genes in the oocyte [12]. We also demonstrated that this post-fertilization methylation activity existed at the endogenous *H19* ICR as well [12]. Hence, we suggest that the maintenance of imprinted methylation during pre-implantation development is governed by de novo methylation activity mediated by paternal allele- and sequence-specific, yet DNA methylation-independent, DNA-binding factors. This notion is supported by our findings that a 118-bp sequence lacking any CpG motif (Additional file 3: Fig. S3A) is necessary and sufficient for post-fertilization imprinted DNA methylation. It is unlikely that ZFP57 and/or ZFP445 act through the 118-bp sequence, as they recognize methylated DNA. In support of this hypothesis, we also failed to detect binding of the ZFP57 protein to the 118-bp sequence in gel-shift assays [15].

How do these two seemingly independent mechanisms collaborate to maintain methylation imprinting? As mentioned earlier, deletion of the 116-bp sequence from the endogenous *H19* ICR resulted in reduced methylation of this locus during the pre-implantation period, suggesting that the methylation maintenance activity of ZFP57/ZFP445 during this period is insufficient. Due to predominant genome-wide demethylation activity during this period, additional maintenance involving the 118-bp sequence of the *H19* ICR may be necessary. By contrast, Takahashi et al. reported that almost all methylation was lost at the endogenous ICRs in *Zfp57*/*Zfp445* double mutant mice by around E11.5. Furthermore, we previously suggested that post-fertilization de novo methylation activity of the *H19* ICR disappears sometime during early embryogenesis [12]. These results together imply that ZFP57/ZFP445-dependent activity is the sole mechanism responsible for post-implantation methylation maintenance at the *H19* ICR.

We can envision two compatible mechanisms by which the 118-bp sequence could contribute to de novo methylation at the paternally inherited *H19* ICR soon after fertilization (Fig. 8f). First, since histones rather than protamine are retained at the *H19* locus in sperm [24, 25], the 118-bp sequence might be involved in the establishment of epigenetic modifications during spermatogenesis, either as a binding site for specific histone modification enzymes or as the deposition site for the marks. Such a non-methylation mark would then be utilized to distinguish the parental origin of the alleles and somehow be translated into differential DNA methylation after fertilization. Second, the sequence might act as a scaffold for recruitment of de novo DNA methyltransferases in pre-implantation embryos. Specific DNA-binding factors, which have not yet been identified, might recognize the 118-bp sequence associated with allele-discriminating signatures and recruit de novo DNA methyltransferases (i.e, Dnmt3A and 3L) in early embryos. Identification of the factors that bind the 118-bp sequence should provide insight into the molecular mechanism of post-fertilization, allele-specific methylation at the *H19* ICR.

IG (intergenic)-DMR of the *Dlk1*-*Dio3* imprinted domain is one of the three ICRs that acquires DNA methylation in sperm. Recent work showed that deletion of a tandem repeat sequence (300–400 bp) from the paternal IG-DMR caused loss of methylation only after the fertilization period [26]. Since the murine repeat array of the IG-DMR contains several consensus binding sites for Zfp57, it is conceivable that the phenotype was caused by a loss of Zfp57-dependent methylation maintenance. Curiously, however, the consensus motifs are not present in the repeat arrays of the human and sheep sequences [27]. Therefore, it is conceivable that a Zfp57-independent mechanism that is common to both *H19* ICR and IG-DMR is operating at these paternal gDMRs through the 118-bp and the repeat array sequences, respectively, although they do not share significant sequence homology. In addition, the corresponding region of the human *H19* ICR sequence (hIC1) is not strongly similar to the mouse 118-bp sequence, and Hur et al. failed to recapitulate paternal methylation of the hIC1 (4.8 kb) when knocked into the mouse *Igf2/H19* locus [28]. It remains an open question whether the mechanism of post-fertilization methylation maintenance we found in the mouse *H19* ICR is conserved in other mammals, especially in humans, and whether it is also employed at other imprinted loci.

## Conclusions

We showed that the 118-bp region of the *H19* ICR is responsible for post-fertilization acquisition of DNA methylation at the paternal ICR in both transgenic and endogenous loci. The reconstituted LCb118 fragment not only exhibited methylation dynamics identical to that of the wild-type *H19* ICR fragment in the transgenic context, but also recapitulated imprinted methylation and imprinted expression of the *Igf2/H19* genes when used to replace the endogenous *H19* ICR. These results demonstrated that the imprinted status in the mouse genome can be generated by an artificial fragment that includes a limited number of *cis* elements.

## Methods

### Mice

#### Generation of YAC-TgM

##### Preparation of a series of 5′-deletion fragments of the *H19* ICR

Two oligonucleotides, 5′-GATCCCGGGGTACCAGATCTTTTCTGCAGTGTAC-3′ and 5′-ACTGCAGAAAAGATCTGGTACCCCGG-3′ (restriction enzyme sites are underlined), were annealed (generating BamHI–KpnI–BglII–PstI sites) and ligated to BamHI/KpnI-digested pBluescriptII/KS(+) to generate pBS-BKpBgPs, by which the KpnI site in the multicloning site was removed. The “ICR432″ fragment, prepared from the pHS1/loxPw+/*ICR* plasmid [11] by digestion with KpnI [at nucleotide 1777 (AF049091; GenBank)] and BamHI (at nucleotide 3696), was ligated to the BamHI/KpnI-digested pBS-BKpBgPs to generate the pBSK/ICR432 plasmid.

A series of 5′-deletion fragments of 5′ portion of the *H19* ICR sequence [nucleotides 1126 (BglII)-1543 (EcoRI)-1776 (KpnI) (AF049091; GenBank)] was generated by PCR using a common 3′ primer: 5′-GGCGGATCCCGGggtaccagcctagaaaatgc-3′ (BamHI and KpnI sites underlined) and a variable 5′ primers as follows:

- 5′del_fr-3A9, 5′-AAAAACTGCAGGATCCagatctagctctatccca-3′ (PstI–BamHI–BglII);
- 5′del_fr-3A8, 5′-AAAAACTGCAGGATCCaagctttcctgctcactg-3′ (PstI–BamHI–*Hin*dIII);
- 5′del_fr-3A7, 5′-AAAAACTGCAGGATCCacatagcagtgctgtgac-3′ (PstI–BamHI);
- 5′del_fr-3A6, 5′-AAAAACTGCAGGATCCccatgtaagtgtgttctg-3′ (PstI–BamHI);
- 5′del_fr-3A5, 5′-AAAAACTGCAGGATCCcctgagttaaaaccgaga-3′ (PstI–BamHI);
- 5′del_fr-3A4, 5′-AAAAACTGCAGGATCCaaaaaggttggtgagaaa-3′ (PstI–BamHI);
- 5′del_fr-3A3, 5′-AAAAACTGCAGGATCCcacttacacccaggactc-3′ (PstI–BamHI) and
- 5′del_fr-3A2, 5′-AAAAACTGCAGGATCCgaattctgcaaggagacc-3′ (PstI–BamHI–EcoRI).

Sequences in lower case letter are complementary to the *H19* ICR sequences. Resultant fragments were digested with KpnI/PstI and individually ligated to KpnI/PstI-digested pBSK/ICR432 to generate pBSK/ICR4321/5′del-9–2. Insert fragments (5′del-9 ~ 2) were released by digestion with BamHI and used for following construction steps.

##### Preparation of co-placement yeast targeting vectors for 5′-del mutants

The co-placement target vector, pHS1/loxP-5171-B-2272-5171-G-2272, carrying a human β-globin HS1 fragment [nucleotides 13,299–14,250 (HUMHBB; GenBank)], in which 5′-loxP5171-BamHI-loxP2272-loxP5171-BglII-loxP2272-3′ sequences are introduced into the *Hin*dIII site [at nucleotide 13,769 in HUMHBB], was described elsewhere [14].

One of the 5′-deletion fragments (5′del-9, 7, 5 or 3) was ligated with BglII-digested pHS1/loxP-5171-B-2272-5171-G-2272 to generate pCop5B25 (5′del-9, 7, 5 or 3)2, respectively. The resultant plasmid was digested with BamHI and ligated with another fragment, 5′del-8, 6, 4 or 2, to generate pCop5 (5′del-8, 6, 4 or 2)25 (5′del-9, 7, 5 or 3)2, respectively. In each cloning step, the correctness of DNA construction was confirmed by DNA sequencing.

##### Preparation of the LCb118 fragment

Two DNA fragments were generated by PCR using either the murine *H19* ICR DNA as a template and a set of primers: 5′del_fr-3A8G + B, 5′-CTAGAGATCTGGATCCAAGCTTTCCTGCTCACTG-3′ (BglII, BamHI and *Hin*dIII sites underlined) and ICRcore-118-3A, 5′-TTGAATTCACCATGGCCCTTTAGCC-3′ (EcoRI), or the λ DNA as a template and a set of primers: Lambda-5S, 5′-CGGAATTCaaaagtggggaagtgagt-3′ (EcoRI; λ sequences in lower case letters) and LS5, 5′-TATTCTCGAG ACGCGTTTTGCTGCCACCACGCGGCAACtaggtgttttaactcgtg-3′ (XhoI, MluI and CTCF binding sites are underlined; λ sequences in lower case letters). Resultant fragments were digested with BglII/EcoRI and EcoRI/MluI, respectively, linked together at their EcoRI ends to generate 5′ segment of the LCb118 sequence. Preparation of λ + CTCF + b (LCb) sequences were described elsewhere [14]. The LCb fragment, released by BamHI digestion was blunt-ended and ligated with BglII linker (pCAGATCTG). 3′ segment of this fragment, carrying CTCF sites 2 to 4, was released by MluI/BglII digestion and linked to 5′ segment of the LCb118 sequence (BglII–MluI fragments, described above) to generate an LCb118 BglII fragment.

##### Preparation of co-placement yeast targeting vector for LCb/LCb118 sequences

The LCb fragment was inserted into BamHI site of pCop5B25G2 to generate pCop5[LCb]25G2. The resultant plasmid was digested with BglII and ligated with the LCb118 fragment to generate pCop5[LCb]25[LCb118]. In each cloning step, the correctness of DNA construction was confirmed by DNA sequencing.

##### Generation of YAC-TgM

The targeting vectors were linearized with SpeI [at nucleotide 13,670 in HUMHBB] and used to mutagenize the human β-globin YAC (A201F4.3) [29]. Successful homologous recombination in yeast was confirmed by Southern blot analyses with several combinations of restriction enzymes and probes.

Purified YAC DNA was microinjected into fertilized mouse eggs from CD1 (ICR) (for generation of 5′-del TgM) or C57BL/6 J (for generation of LCb/LCb118 TgM) mice. Tail DNA from founder offspring was screened first by PCR, followed by Southern blotting. Structural analysis of the YAC transgene was performed as described elsewhere [29, 30]. TgM ubiquitously expressing cre recombinase [31] or TgM carrying Zp3-Cre gene (Jackson Laboratory; [32]) were mated with parental YAC-TgM lines to generate sublines (i.e., each carrying one of the test fragments). Successful Cre-loxP recombination was confirmed by Southern blotting.

#### Deletion of 116-bp sequences from transgenic and endogenous *H19* ICR in mouse by CRISPR/Cas9 genome editing

Two sets of oligonucleotides were annealed and inserted at the BbsI site of the pX330 (plasmid #42230; Addgene) [33] to generate Cas9/sgRNA expression vectors. For the 5′ border: 5′-caccGAGTGAGCAGGAAAGCTTCCT-3′ and 5′-aaacAGGAAGCTTTCCTGCTCACTC-3′; and for the 3′ border: 5′-caccGAACACACTTACATGGCACCA-3′ and 5′-aaacTGGTGCCATGTAAGTGTGTTC-3′ (overhanging nucleotides are shown in lowercase letters). The plasmids were microinjected into the pronuclei of fertilized eggs of ICR/β-globin TgM (CD-1 background; [11]). Tail DNA from founder offspring was screened by PCR and sequencing. For the establishment of knockout mice of the endogenous *H19* ICR, founder offspring was backcrossed with wild-type C57BL/6J mice for at least five generations.

#### Generation of LCb/LCb118 knock-in mice

*Targeting vector construction*

[Backbone vector]

Two oligonucleotides, SPAN-5S: 5′-GGCCGCACTAGTTTAATTAAGGCGCGCCACCGGTGGCGCC-3′ and SPAN-3A: 5′-TCGAGGCGCCACCGGTGGCGCGCCTTAATTAAACTAGTGC-3′, were annealed and ligated with NotI/XhoI-digested pMCDT-A(A + T/pau) (Analytical Biochemistry, 1993, 77–86). The resultant plasmid, pMCDT-A/SPAN carried NotI–SpeI–PacI–AscI–AgeI–NarI–XhoI multi-cloning sites. Then, a bovine growth hormone (bGH) polyA fragment was generated by PCR using the pMC1neo-polyA (Stratagene) as a template and a set of primers, bGH(XbaI)-5S: 5′-GCTCTAGACTGTGCCTTCTAGTTGCCA-3′ (XbaI site is underlined) and bGH(SpeI)-3A: 5′-CCATAGAGCCCACTAGTTCCCCAGCATGCC-3′ (SpeI). The resultant fragment was digested by XbaI/SpeI and introduced into SpeI site of the pMCDT-A/SPAN to generate pMCDT-A-pA/SPAN.

[3′-homology fragment]

To generate ploxP2272-3′homology fragment, two oligonucleotides, Bas2272G-S: 5′-CCGGAGGCGCGCC*ATAACTTCGTATAGGATACTTTATACGAAGTTAT*A-3′ and Bas2272G-AS: 5′-GATCT*ATAACTTCGTATAAAGTATCCTATACGAAGTTAT*GGCGCGCCT-3′ (loxP2272 sequences are italicized and AscI sites underlined), were annealed and ligated with BspEI/BglII-digested p3′hom (BspEI–SpeI) [12]. A loxP2272-3′-homology (from nt 135,261 to 138,910) fragment was released from the plasmid by digestion with AscI and NarI, and ligated with AscI/NarI-digested pMCDT-A-pA/SPAN to generate pMCDT-A-pA/SPAN + 3′hom.

[5′-homology fragment]

To generate p5′hom-loxP5171 fragment, two oligonucleotides, B5171AsB-S: 5′-GATCC*ATAACTTCGTATAGTACACATTATACGAAGTTAT*GGCGCGCCT-3′ and B5171AsB-AS: 5′-CCGGAGGCGCGCC*ATAACTTCGTATAATGTGTACTATACGAAGTTAT*G-3′ (loxP5171 sequences are italicized and AscI sites underlined) were annealed and ligated with BamHI/BspEI-digested p5′hom (SpeI–BspEI) [12]. The murine *H19* ICR BglII fragment (from nt 131,758 to 132,889; AC013548.13) was ligated with BglII/BamHI-digested p5′hom-loxP5171 to generate p5′hom(B)-loxP5171. Then, another *H19* ICR BglII fragment (from nt 126,424 to 131,758; AC013548.13) was ligated with BglII-digested p5′hom(B)-loxP5171 to generate p5′-homology-loxP5171. A 5′-homology (from nt 125,932 to 132,889)-loxP5171 fragment was released from the resultant plasmid by digestion with SpeI and AscI, and ligated with SpeI/AscI-digested pMCDT-A-pA/SPAN + 3′hom to generate pMCDT-A-pA/SPAN + 3′hom + 5′hom.

##### Preparation of CRISPR/guide RNA expression and LCb/LCb + 118 donor plasmids

Two oligonucleotides, 5′-caccGTGGTTCATTTGCATTTCGA-3′ and 5′-aaacTCGAAATGCAAATGAACCAC-3′, were annealed and ligated with BbsI-digested pX459 (addgene) to generate pX459-H19-inv-2.

Two oligonucleotides, SABAK-S: 5′-CGGCGCGCCGGATCCGGCGCGCCGGTAC-3′ and SABAK-AS: 5′-CGGCGCGCCGGATCCGGCGCGCCGAGCT-3′ were annealed and ligated with KpnI/SacI-digested pBluescriptII KS(+) to generate pBSIIKS + SABAK that carries SacI–AscI–BamHI–AscI–KpnI multi-cloning sites. The LCb and LCb118 fragments were released from pHS1/loxP5171-LCb-2272-5171-LCb118(+B)-2272 (ref.) by BamHI digestion, separately introduced into BamHI site of the pBSIIKS + SABAK, and recovered by digestion with AscI of the resultant plasmids. The XbaI fragment, harboring 5′-homology (1159 bp)-loxP5171-AscI site-loxP2272-3′-homology (1201 bp) sequences, was excised from the pMCDT-A-pA/SPAN + 3′hom + 5′hom mentioned above and introduced into XbaI site of the pBluescriptII KS(+) to generate pBSII/3′hom + 5′hom. The LCb and LCb118 AscI fragments were introduced into AscI site of the pBSII/3′hom + 5′hom to generate donor plasmids, pBSII/3′hom + 5′hom/LCb and pBSII/3′hom + 5′hom/LCb118, respectively.

##### CRISPR/Cas9-assisted homologous recombination in ES cells

B6 J-S1 ES cells derived from C57BL/6J mouse strain [34] were maintained in DMEM High Glucose (containing l-glutamine and sodium pyruvate, No. 11995, ThermoFisher Scientific) supplemented with 15% knockout serum replacement (KSR; No. 10828-028, Invitrogen, San Diego, CA), 0.1 mM nonessential amino acids, penicillin (50 U/ml)-streptomycin (50 µg/ml), 0.1 mM 2-mercaptoethanol, 1% FBS, 1000 units/ml leukemia inhibitory factor (No. ESG1107, Chemicon, Temecula, CA), 1 µM PD0325901 (No. 162-25291, Wako) and 3 µM CHIR99021 (No. 034-23103, Wako).

Eight hours before transfection, ES cells (1.7 × 10^5^ cells/well) were seeded in 6-well plate. CRISPR/guide RNA expression (1.25 µg), as well as the LCb or LCb + 118 donor plasmids (1.25 µg) were transfected into cells by Lipofectamine LTX (Thermo Fisher Scientific). Puromycin selection (1 µg/ml, No. ant-pr-1, Invivogen) started at 20 h after transfection and continued for another 45 h. Cells were then stripped, made single-cell suspension and seeded onto feeder-cell plates in the medium without puromycin. After 3 days culture, colonies were picked up, expanded and homologous recombination event was checked by PCR and Southern blotting with several combinations of restriction enzymes and probes. Chimeric mice were generated by a coculture method using eight-cell embryos from CD1 mice (ICR, Charles River Laboratories). Chimeric males were bred with B6J females, and germ line transmission of the mutant allele was identified by Southern blot analysis.

#### Preparation of embryos

Female mice were super-ovulated via injection of pregnant mare serum gonadotropin, followed by human chorionic gonadotropin (hCG) (47-48 h interval). Two-cell embryos were flushed from oviducts by M2 medium at 44 h after hCG injection, and then washed by PBS. Embryos at E3.5 (blastocysts), E12.5, and E18.5 were obtained by natural mating.

#### DNA methylation analysis by southern blotting

Genomic DNA extracted from tail somatic cells was first digested by *Eco*T22I (for analysis of the 5′-deleted transgenic *H19* ICR) or BamHI (for analysis of the 116-bp deleted transgenic *H19* ICR or the LCb and LCb118 transgenes) and then subjected to the methylation-sensitive enzymes BstUI or *Hha*I. Following size separation in agarose gels, Southern blots were hybridized with α-^32^P-labeled probes and subjected to X-ray film autoradiography.

#### DNA methylation analysis by bisulfite sequencing

Pre-implantation embryos were embedded in agarose beads and treated with sodium bisulfite as described previously [10]. Genomic DNA extracted from adult male sperm or the tail tips of ~ 1-week-old animals was treated with sodium bisulfite using the EZ DNA Methylation Kit (Zymo Research). Sperm and tail tip DNA was digested with XbaI prior to the treatment. Subregions of the transgenic *H19* ICR and the transgenic, as well as the knock-in LCb/LCb118 fragments were amplified by nested PCR, while those of the endogenous *H19* ICR with the 116-bp-deletion were amplified by single-round PCR. The PCR products were subcloned into the pGEM-T Easy vector (Promega) for sequencing analyses. PCR primers are listed in Tables 1 and 2.

**Table 1:** Primer sets for bisulfite sequencing analysis

Regions analyzed	Allele	PCR round		5′ primer	3′ primer
I	del-6 to del-8 Tg-5′ICR-KO(116)	Nested PCR	1st	LCR-MA-5S1	ICR-MA-3A15
			2nd	ICR-MA-5S4	ICR-MA-3A14
II	del-6 to del-8	Nested PCR	1st	ICR-MA-5S13	BGLB-MA-3A2
			2nd	ICR-MA-5S13	ICR-MA-3A2
III	Tg-5′ICR-KO(116)	Nested PCR	1st	ICR-MA-5S13	BGLB-MA-3A2
			2nd	ICR-MA-5S13	ICR-MA-3A26
III′	endo-5′ICR-KO(116)	Single-round PCR		ICR-MA-5S13	ICR-MA-3A26
IV	Tg-LCb Tg-LCb118	Nested PCR	1st	lambda-MA-5S4	lambda-MA-3A2
			2nd	lambda-MA-5S1	lambda-MA-3A3
V	Tg-LCb Tg-LCb118	Nested PCR	1st	lambda-MA-5S5	lambda-MA-3A7
			2nd	lambda-MA-5S6	lambda-MA-3A8
VI	Tg-LCb	Nested PCR	1st	lambda-MA-5S7	BGLB-MA-3A6
			2nd	lambda-MA-5S8	BGLB-MA-3A2
VII	Tg-LCb118	Nested PCR	1st	lambda-MA-5S7	BGLB-MA-3A6
			2nd	lambda-MA-5S8	BGLB-MA-3A2
VIII	Endo-LCb Endo-LCb118	Nested PCR	1st	lambda-MA-5S7	ICR-MA-3A21
			2nd	lambda-MA-5S8	lambda-MA-3A1

**Table 2:** PCR primer sequences for bisulfite sequencing analysis

	Name	Sequences
5′ primer	ICR-MA-5S4	5′-GAATTTGGGGTATTTAAAGTTTTG-3′
	ICR-MA-5S13	5′-GGTGATTTATAGTATTGTTATTTG-3′
	LCR-MA-5S1	5′-TATAGATGTTTTAGTTTTAATAAG-3′
	lambda-MA-5S1	5′-ATTAGTAAGAAGATAGTAGTGATG-3′
	lambda-MA-5S4	5′-TTAAGTTTTGTGTGTTATTTATTA-3′
	lambda-MA-5S5	5′-GTTAAAAAGAAGAAGTAAGTATTT-3′
	lambda-MA-5S6	5′-GTGAAAGTATTGATTATTATGTTA-3′
	lambda-MA-5S7	5′-GAGGTTTATTTGTATTTATTTTTGTT-3′
	lambda-MA-5S8	5′-TATTTTTTAGTAGTATTGTAAGAGGT-3′
3′ primer	ICR-MA-3A2	5′-AACAATACTAAATCTACCTAAAAC-3′
	ICR-MA-3A14	5′-AAAACATAAAAACTATTATATACA-3′
	ICR-MA-3A15	5′-ACCAACCAATATAACTCACTATAA-3′
	ICR-MA-3A21	5′-ACTTACTACCCTCATTATACTTT-3′
	ICR-MA-3A26	5′-CAAATTAACAAAAACATACCTAACT-3′
	BGLB-MA-3A2	5′-TTCTAACCCCACAAAAATTTATTC-3′
	BGLB-MA-3A6	5′-CCAAACCCCCTCTATTTTATATCA-3′
	lambda-MA-3A1	5′-CTAACATTTATCTACATCATACCT-3′
	lambda-MA-3A2	5′-ATACCTTATTTTTTTCTACTACAA-3′
	lambda-MA-3A3	5′-CTAAACTCCAACATATAATAACCC-3′
	lambda-MA-3A7	5′-AACCAAAATTATCTTTTTCTATCT-3′
	lambda-MA-3A8	5′-ACAACATTCTTAAATCCAATATTA-3′

#### Chromatin immunoprecipitation (ChIP) assay

The LCb118 YAC-TgM (2–4 months old) inheriting the transgene either paternally or maternally were made anaemic by phenylhydrazine treatment, and nucleated erythroid cells were collected from their spleens. Livers were obtained from E18.5 embryos inheriting the LCb118 knock-in allele either paternally or maternally. Cells were fixed in PBS with 1% formaldehyde for 10 min at room temperature. Nuclei (2 × 10^7^ cells) were digested with 12.5 units/ml of micrococcal nuclease at 37 °C for 20 min to prepare primarily mono- to di-nucleosome-sized chromatin. The chromatin was incubated with anti-CTCF antibody (D31H2; Cell Signaling Technology) or purified rabbit IgG (Invitrogen) overnight at 4 °C and was precipitated with preblocked Dynabeads protein G magnetic beads (Life Technologies, Carlsbad, CA). Immunoprecipitated materials were then washed extensively and reverse cross-linked. DNA was purified with the QIAquick PCR purification kit (Qiagen, Venlo, the Netherlands) and subjected to qPCR analysis. The endogenous *H19* ICR and *Necdin* sequences were analyzed as positive and negative controls, respectively [35]. PCR primers were reported previously [35].

#### RT-qPCR

Total RNA was recovered from phenylhydrazine treated anaemic adult spleens (1–2 months old) of LCb118 YAC TgM using ISOGEN (Nippon Gene) and converted to cDNA using ReverTra Ace qPCR RT Master Mix with gDNA Remover (TOYOBO). Quantitative amplification of cDNA was performed with the Thermal Cycler Dice (TaKaRa Bio) using TB Green Premix EX TaqII (TaKaRa Bio). PCR primers were reported previously [15].

#### Allele-specific expression analysis

LCb118 knock-in mice (which has a genetic background of *Mus musculus domesticus*) were mated with wild-type JF1 mice (which was provided by the RIKEN BRC through the National Bio-Resource Project of the MEXT, Japan, and of which genome is basically from *Mus musculus molossinus*) to distinguish parental origin of the alleles in the offspring. Total RNA from livers of E12.5 or E18.5 embryos was converted to cDNA as described above. PCR was performed using AmpliTaq Gold 360 and PCR primers listed in Table 3 with (E12.5 cDNA) or without (E18.5 cDNA) α-^32^P-dCTP. The amplified products were digested with BstUI or Cac8I, in order to discriminate the parental origin of the transcripts. The sites were also introduced into primer sequence so that complete digestion of PCR products can be concomitantly monitored.

**Table 3:** Primer sequences for RT-PCR

Gene	Primer name	Sequences
*Igf2*		
5′ primer	mIgf2-5S2	5′-TCTGTGCGGAGGGGAGCTTGTT-3′
3′ primer	mIgf2-3A2	5′-CAGCACTCTTCCGCGATGCCAC-3′
*H19*		
5′ primer	mH19-5S2	5′-CGGTGTGATGGAGAGGACAGAAG-3′
3′ primer	mH19-3A2	5′-CCAGAGAGCAGCAGGCAAGTGTTAG-3′
*Gapdh*		
5′ primer	mGAPDH-5S	5′-AAAATGGTGAAGGTCGGTGTG-3′
3′ primer	mGAPDH-3A	5′-TGAGGTCAATGAAGGGGTCGT-3′

## Supplementary information

**Additional file 1: Figure S1.** Generation and structural analysis of YAC-TgM carrying the 5′-truncated *H19* ICR fragments. **(A)** Schematic representation of the YAC transgenes. The positions of the β-like globin genes (filled boxes) are shown relative to the locus control region (LCR, gray box). SfiI restriction enzyme sites are located 5′ to the LCR, within the LCR, and in the right arm of the YAC. Probes (filled rectangles) used for long-range structural analyses shown in panels (B), and the expected restriction enzyme fragments and their sizes are shown. The enlarged map shows the detailed structure of the del-8/9, 6/7, 4/5, and 2/3 fragments inserted between the LCR and the λ-globin gene. The positions of loxP5171 and loxP2272, inserted for employing the co-placement strategy, are indicated as solid and open triangles, respectively. **(B)** Long-range structural analysis of the transgenes in the YAC-TgM. DNA from thymus cells was digested with SfiI in agarose plugs and separated by pulsed-field gel electrophoresis, and Southern blots were hybridized separately to probes shown in (A). **(C)** In vivo Cre-loxP recombination in the parental del-8/9 transgene generates either del-8 or del-9 daughter transgenes. Positions of BamHI (B) restriction enzyme sites, and the expected restriction enzyme fragments and their sizes are shown. For example, if recombination occurs between the loxP5171 sites (solid triangles), no further recombination can occur because one of the loxP2272 sites (open triangles) is concomitantly deleted. The probe used for Southern blot analysis in (D) was shown as filled rectangles. The other TgM sub-lines were also generated by the same strategy. **(D)** Tail DNA from each YAC-TgM sublines was digested with BamHI and separated on agarose gels, and Southern blots were hybridized to the probe shown in (C).

**Additional file 2: Figure S2.** DNA methylation status of the 5′-truncated *H19* ICR fragments in somatic cells of YAC-TgM. **(A)** Partial restriction enzyme maps of the endogenous *H19* locus and the β-globin YAC transgenes with the inserted 5′-truncated *H19* ICR fragments. Methylation-sensitive BstUI sites in the EcoT22I (ET) fragments are displayed as vertical lines beneath each map. The ICR43 probe used for Southern blot analysis in (B–I) is shown as a filled rectangle. B; BamHI, G; BglII, Sa; SacI sites. **(B–I)** DNA methylation status of the *H19* ICR fragment in somatic cells of the YAC-TgM that inherited the transgenes either paternally (pat.) or maternally (mat.). Tail DNA was digested with EcoT22I and then BstUI, and the blot was hybridized with the ICR43 probe shown in (A). endo.; endogenous locus, Tg; transgene. Asterisks indicate the positions of parental or methylated, undigested fragments.

**Additional file 3: Figure S3.** Introduction of 116-bp deletional mutations within the transgenic or endogenous *H19* ICR in mice by CRISPR/Cas9 genome editing. **(A)** Sequence alignment of wild-type and the mutant *H19* ICRs. Protospacer-adjacent motif (PAM) and gRNA sequences are shaded and underlined, respectively. Cleavage sites predicted by PAM locations (arrowheads), as well as the end positions of del-5-9 fragments are shown. **(B)** Partial restriction enzyme maps of the endogenous *H19* locus and the β-globin YAC transgene carrying the *H19* ICR fragment with the 116-bp deletion. Methylation-sensitive HhaI sites in the BamHI (B) fragments are displayed as vertical lines beneath each map. The probe used for Southern blot analysis in (C) is shown as a filled rectangle. B; BamHI, G; BglII, H; HindIII, Sa; SacI sites. **(C)** DNA methylation status of the mutant *H19* ICR fragment in somatic cells of the YAC-TgM that inherited the transgene either paternally (pat.) or maternally (mat.). Tail DNA was digested with BamHI and then HhaI, and the blot was hybridized with the probe shown in B. endo.; endogenous locus, Tg; transgene. Asterisks indicate the positions of parental or methylated, undigested fragments.

**Additional file 4: Figure S4.** Generation and structural analysis of YAC-TgM carrying the LCb and LCb118 fragments. **(A)** Structure of the 150-kb human β-globin locus YAC. The LCR and β-like globin genes are denoted as gray and filled boxes, respectively. The enlarged map shows tandemly arrayed LCb and LCb118 fragments, inserted 3′ to the LCR for employing co-placement strategy. The positions of loxP5171 and loxP2272 are indicated as solid and open triangles, respectively. The expected SfiI restriction enzyme fragments (thick lines) and probes (filled rectangles) used in (B) are shown. **(B)** Long range structural analysis of the LCb-LCb118 YAC transgene. DNA from thymus cells was digested with SfiI in agarose plugs and separated by pulsed-field gel electrophoresis, and Southern blots were hybridized separately to probes. **(C)** In vivo Cre-loxP recombination to derive LCb or LCb118 TgM. Tail DNA from parental and daughter YAC-TgM sublines was digested with KpnI and analyzed by Southern blotting using the probe.

**Additional file 5: Figure S5.** DNA methylation status of the LCb and LCb118 fragments in somatic cells of YAC-TgM. **(A and C)** Partial restriction enzyme maps of the β-globin YAC transgenes with the inserted LCb (A) or LCb118 (C) fragments. Methylation-sensitive BstUI sites in BamHI fragments are displayed as vertical lines beneath each map. **(B and D)** DNA methylation status of the LCb (B) or LCb118 (D) fragments in tail somatic cells of the YAC-TgM. Tail genomic DNA was digested with BamHI alone (B) or BamHI + BstUI (B + BstUI) and the Southern blots were hybridized with the probe shown in the maps (A and C). Asterisks indicate the positions of parental or methylated, undigested fragments. ID numbers of individuals inheriting the transgene maternally and paternally are highlighted in pink and blue colors, respectively. In the pedigree, male and female individuals are represented as rectangles and circles, respectively. Filled, gray, or open symbols indicate hyper-, partially-, or hypo-methylated status of LCb or LCb118 fragment in each TgM, which was independently determined by visual examination of the Southern blot results by three individuals. Tail DNA from underlined animals (in the pedigree) was pooled according to the transgene’s parental origin and analyzed by bisulfite sequencing in Fig. 5b, c. Testis samples in Additional file 6: Fig. S6 were obtained from male individuals marked by stars.

**Additional file 6: Figure S6.** DNA methylation status of the LCb and LCb118 fragments in testes. Testis genomic DNA from adult male YAC-TgM was analyzed by Southern blotting as described in the legend to Additional file 5: Fig. S5. Sperm samples were obtained from No. 2037 (LCb, line 890) and 1959 (LCb118, line 890) animals, and methylation status of the transgenes were analyzed by bisulfite sequencing in Fig. 5b, c.

**Additional file 7: Figure S7.** Generation of LCb/LCb118 knock-in mice. **(A)** Targeting strategy at the *H19* locus. Maps of the wild-type allele, the targeting vectors with the LCb or LCb118 fragment replaced by the *H19* ICR, the correctly targeted mutant alleles (endo-LCb and endo-LCb118) are shown from top to bottom. The triangles are the loxP sequences. Probes used for Southern blot analyses in (B) and (C) are shown as filled rectangles. The positions of restriction enzyme sites and expected restriction enzyme fragments with their sizes in each allele are shown nearby. **(B, C)** Following digestion with PstI and separation on agarose gels, genomic DNA from ES clones (B) and mutant mouse tails (C) on Southern blots were hybridized to one of the three probes, 5′EX, L63, or 3′EX.

**Additional file 8: Figure S8.** Monoallelic gene expression pattern is recapitulated in LCb118 knock-in mice. Gene expression analysis of the endo-LCb118 (A) or endo-LCb (B) embryos. In order to distinguish parental origin of the alleles by using SNPs between inbred mouse strains, endo-LCb118 or -LCb hetero-knock-in mice (*H19* ICR/LCb118 or LCb; C57BL/6 J [B6] background) were mated with wild-type mice (*H19* ICR/*H19* ICR; JF1/Msf [JF1]), and offspring was obtained. Total RNA was prepared from livers of E12.5 embryos. *Igf2* and *H19* gene transcripts were amplified by RT-PCR (within logarithmic amplification range) with α-^32^P-labeled dCTP, followed by BstUI or Cac8I digestion, respectively. Parental origin of transcripts was discriminated by allele-specific restriction sites. The sites were also introduced into primer sequence so that complete digestion of PCR products can be concomitantly monitored. *Gapdh* gene transcript was analyzed as control.

**Additional file 9: Figure S9.** Genomic imprinting is recapitulated in LCb118 knock-in mice. DNA methylation status of the paternally inherited endo-LCb118 and -LCb sequences. Genomic DNA were extracted from livers of E12.5 embryos, which were analyzed in Additional file 8: Fig. S8, and used for bisulfite sequencing analysis.

**Additional file 10: Table S1.** Number of pups in endo-LCb or endo-LCb118 knock-in mice.

## Data Availability

The datasets used and analyzed during the current study are available from the corresponding author on reasonable request.
