# Supplementary figures and images for: Recapitulation of gametic DNA methylation and its post-fertilization maintenance with reassembled DNA elements at the mouse Igf2/H19 locus
Source: Epigenetics Chromatin. 2020 Jan 14;13:2. doi: 10.1186/s13072-019-0326-1 (PMC6958606; doi:10.1186/s13072-019-0326-1)

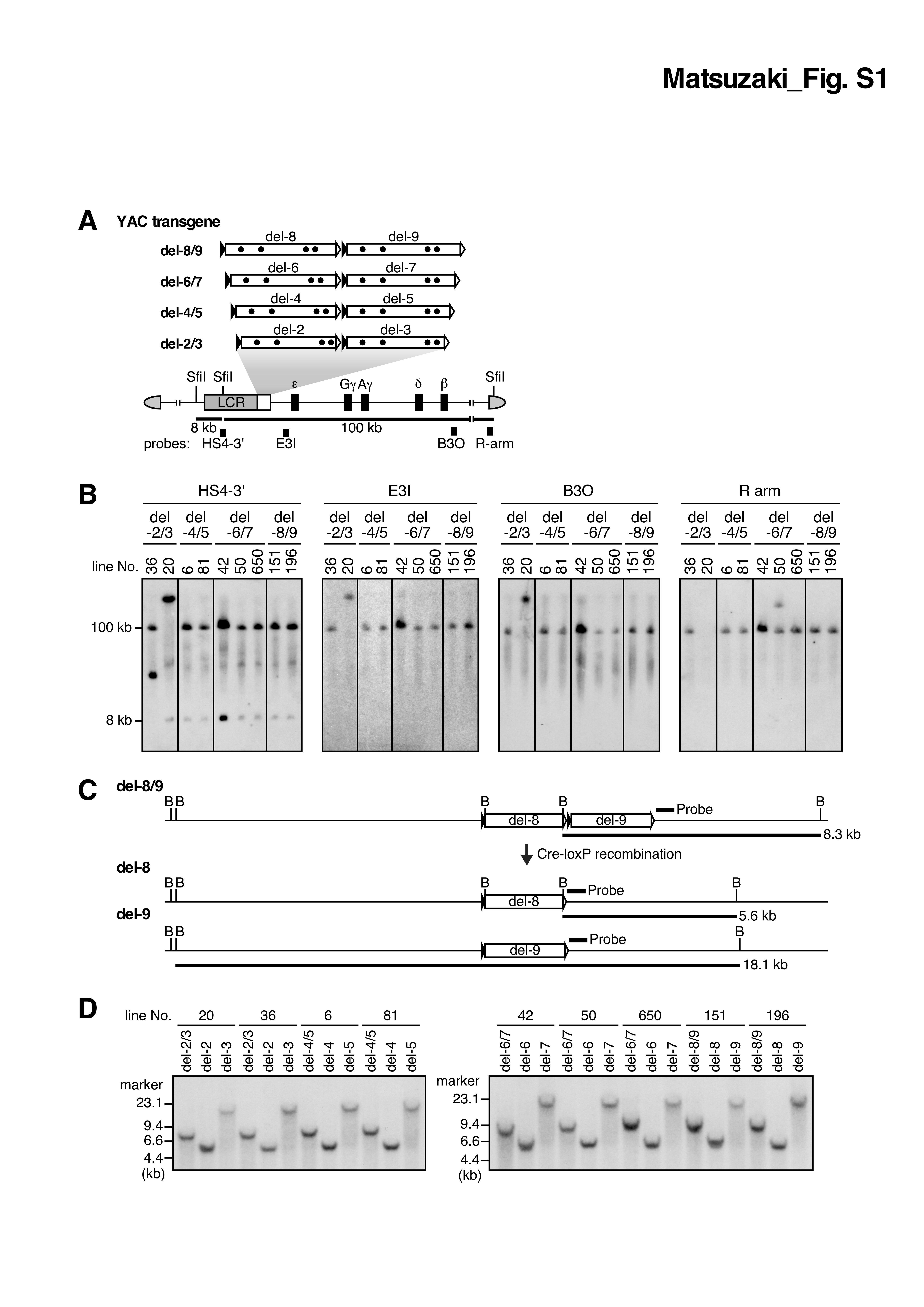

Supplement: Supplementary file 1 — Additional file 1: Figure S1. Generation and structural analysis of YAC-TgM carrying the 5′-truncated H19 ICR fragments. (A) Schematic representation of the YAC transgenes. The positions of the β-like globin genes (filled boxes) are shown relative to the locus control region (LCR, gray box). SfiI restriction enzyme sites are located 5′ to the LCR, within the LCR, and in the right arm of the YAC. Probes (filled rectangles) used for long-range structural analyses shown in panels (B), and the expected restriction enzyme fragments and their sizes are shown. The enlarged map shows the detailed structure of the del-8/9, 6/7, 4/5, and 2/3 fragments inserted between the LCR and the λ-globin gene. The positions of loxP5171 and loxP2272, inserted for employing the co-placement strategy, are indicated as solid and open triangles, respectively. (B) Long-range structural analysis of the transgenes in the YAC-TgM. DNA from thymus cells was digested with SfiI in agarose plugs and separated by pulsed-field gel electrophoresis, and Southern blots were hybridized separately to probes shown in (A). (C) In vivo Cre-loxP recombination in the parental del-8/9 transgene generates either del-8 or del-9 daughter transgenes. Positions of BamHI (B) restriction enzyme sites, and the expected restriction enzyme fragments and their sizes are shown. For example, if recombination occurs between the loxP5171 sites (solid triangles), no further recombination can occur because one of the loxP2272 sites (open triangles) is concomitantly deleted. The probe used for Southern blot analysis in (D) was shown as filled rectangles. The other TgM sub-lines were also generated by the same strategy. (D) Tail DNA from each YAC-TgM sublines was digested with BamHI and separated on agarose gels, and Southern blots were hybridized to the probe shown in (C). [file 13072_2019_326_MOESM1_ESM.tif]

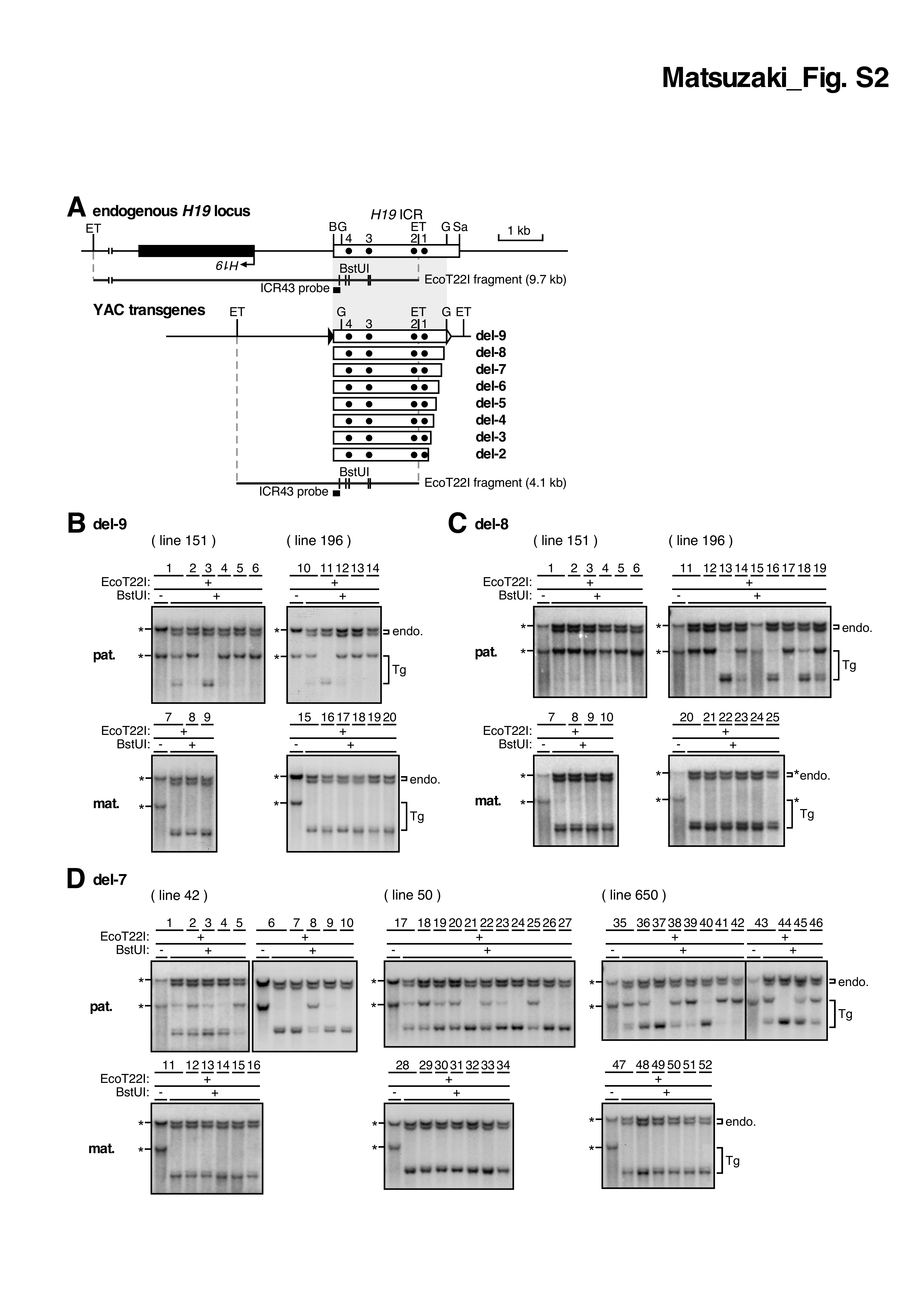

Supplement: Supplementary file 2 — Additional file 2: Figure S2. DNA methylation status of the 5′-truncated H19 ICR fragments in somatic cells of YAC-TgM. (A) Partial restriction enzyme maps of the endogenous H19 locus and the β-globin YAC transgenes with the inserted 5′-truncated H19 ICR fragments. Methylation-sensitive BstUI sites in the EcoT22I (ET) fragments are displayed as vertical lines beneath each map. The ICR43 probe used for Southern blot analysis in (B–I) is shown as a filled rectangle. B; BamHI, G; BglII, Sa; SacI sites. (B–I) DNA methylation status of the H19 ICR fragment in somatic cells of the YAC-TgM that inherited the transgenes either paternally (pat.) or maternally (mat.). Tail DNA was digested with EcoT22I and then BstUI, and the blot was hybridized with the ICR43 probe shown in (A). endo.; endogenous locus, Tg; transgene. Asterisks indicate the positions of parental or methylated, undigested fragments. [file 13072_2019_326_MOESM2_ESM.zip › Fig_S2-1.tif]

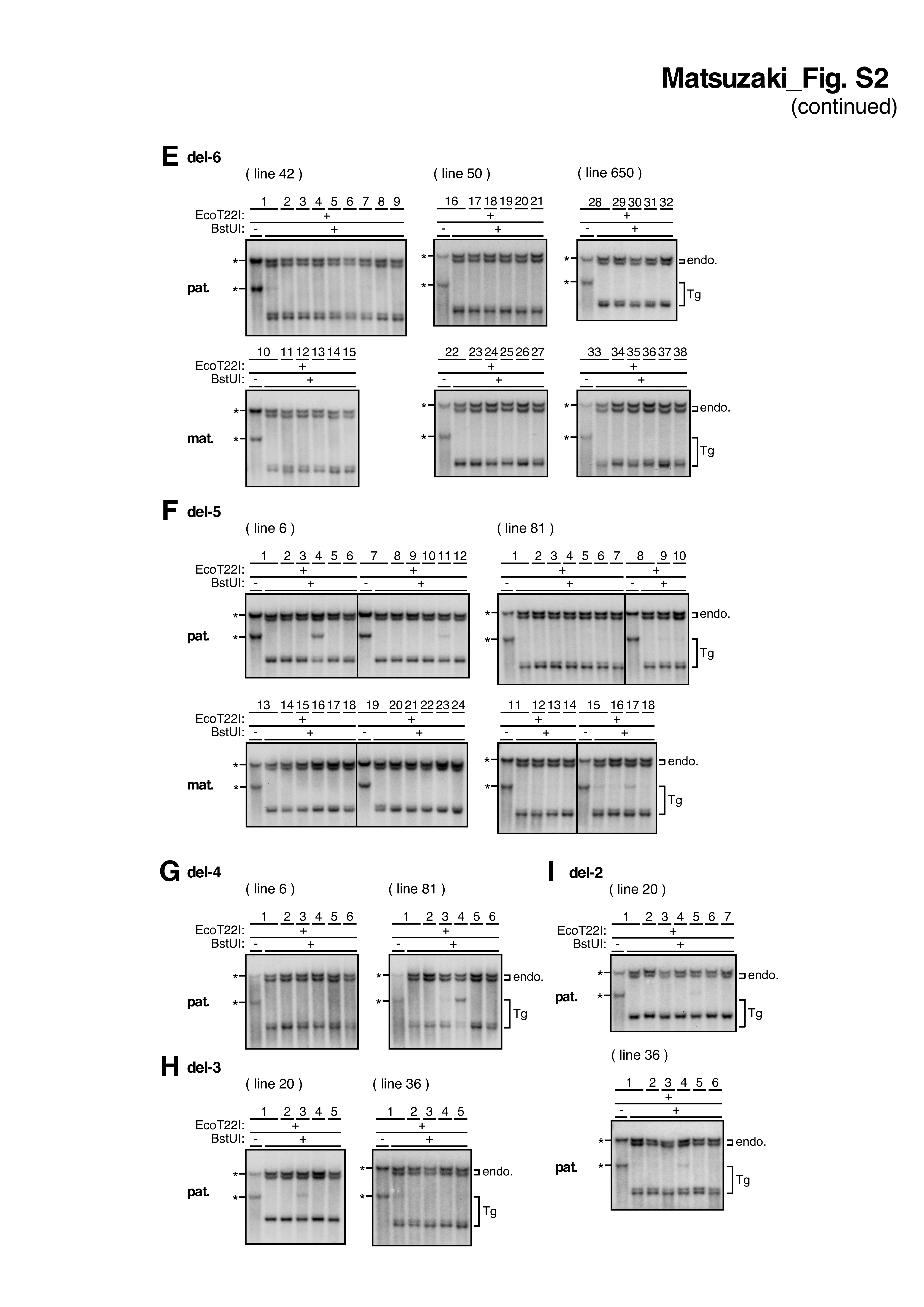

Supplement: Supplementary file 2 — Additional file 2: Figure S2. DNA methylation status of the 5′-truncated H19 ICR fragments in somatic cells of YAC-TgM. (A) Partial restriction enzyme maps of the endogenous H19 locus and the β-globin YAC transgenes with the inserted 5′-truncated H19 ICR fragments. Methylation-sensitive BstUI sites in the EcoT22I (ET) fragments are displayed as vertical lines beneath each map. The ICR43 probe used for Southern blot analysis in (B–I) is shown as a filled rectangle. B; BamHI, G; BglII, Sa; SacI sites. (B–I) DNA methylation status of the H19 ICR fragment in somatic cells of the YAC-TgM that inherited the transgenes either paternally (pat.) or maternally (mat.). Tail DNA was digested with EcoT22I and then BstUI, and the blot was hybridized with the ICR43 probe shown in (A). endo.; endogenous locus, Tg; transgene. Asterisks indicate the positions of parental or methylated, undigested fragments. [file 13072_2019_326_MOESM2_ESM.zip › Fig_S2-2.tif]

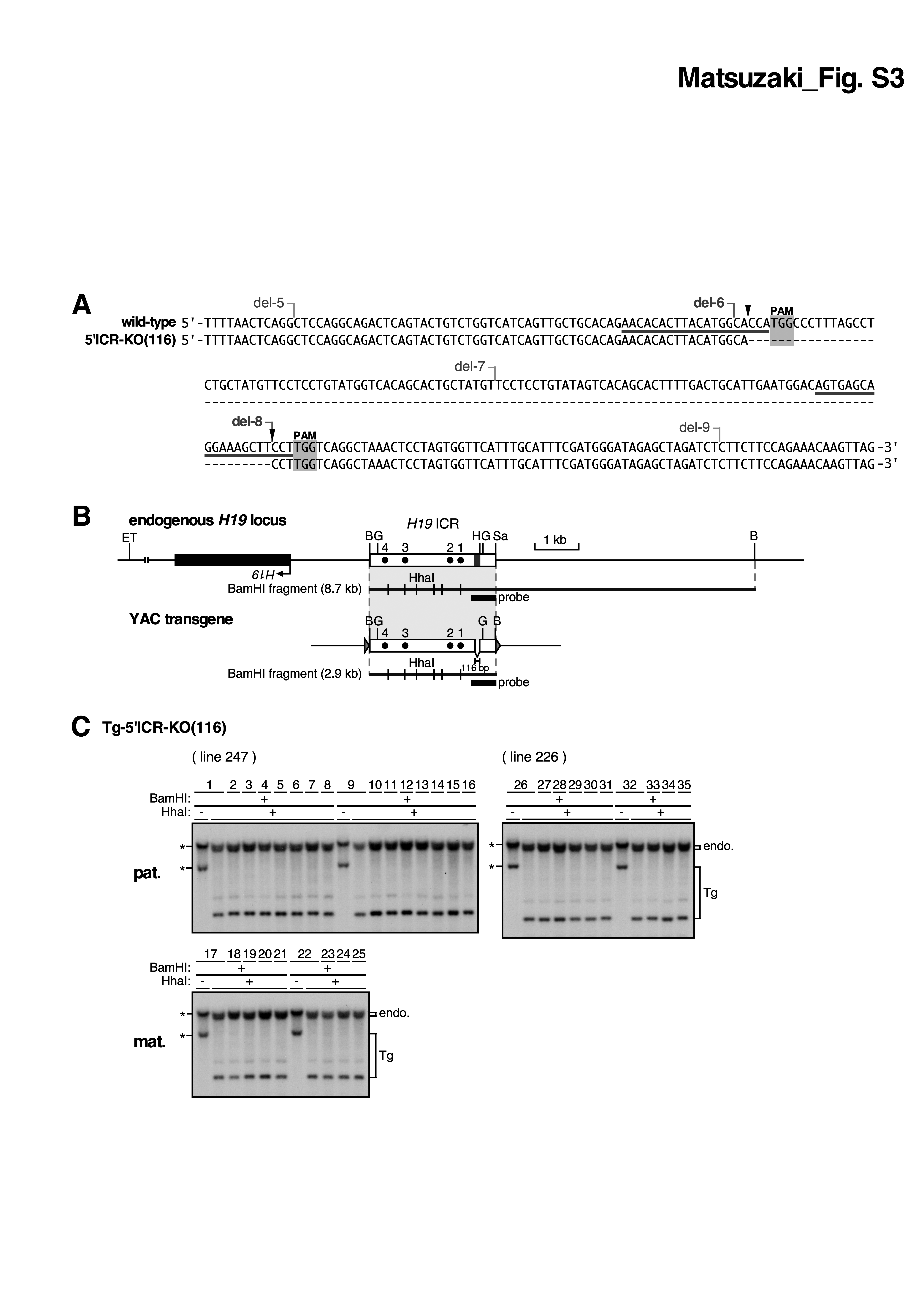

Supplement: Supplementary file 3 — Additional file 3: Figure S3. Introduction of 116-bp deletional mutations within the transgenic or endogenous H19 ICR in mice by CRISPR/Cas9 genome editing. (A) Sequence alignment of wild-type and the mutant H19 ICRs. Protospacer-adjacent motif (PAM) and gRNA sequences are shaded and underlined, respectively. Cleavage sites predicted by PAM locations (arrowheads), as well as the end positions of del-5-9 fragments are shown. (B) Partial restriction enzyme maps of the endogenous H19 locus and the β-globin YAC transgene carrying the H19 ICR fragment with the 116-bp deletion. Methylation-sensitive HhaI sites in the BamHI (B) fragments are displayed as vertical lines beneath each map. The probe used for Southern blot analysis in (C) is shown as a filled rectangle. B; BamHI, G; BglII, H; HindIII, Sa; SacI sites. (C) DNA methylation status of the mutant H19 ICR fragment in somatic cells of the YAC-TgM that inherited the transgene either paternally (pat.) or maternally (mat.). Tail DNA was digested with BamHI and then HhaI, and the blot was hybridized with the probe shown in B. endo.; endogenous locus, Tg; transgene. Asterisks indicate the positions of parental or methylated, undigested fragments. [file 13072_2019_326_MOESM3_ESM.tif]

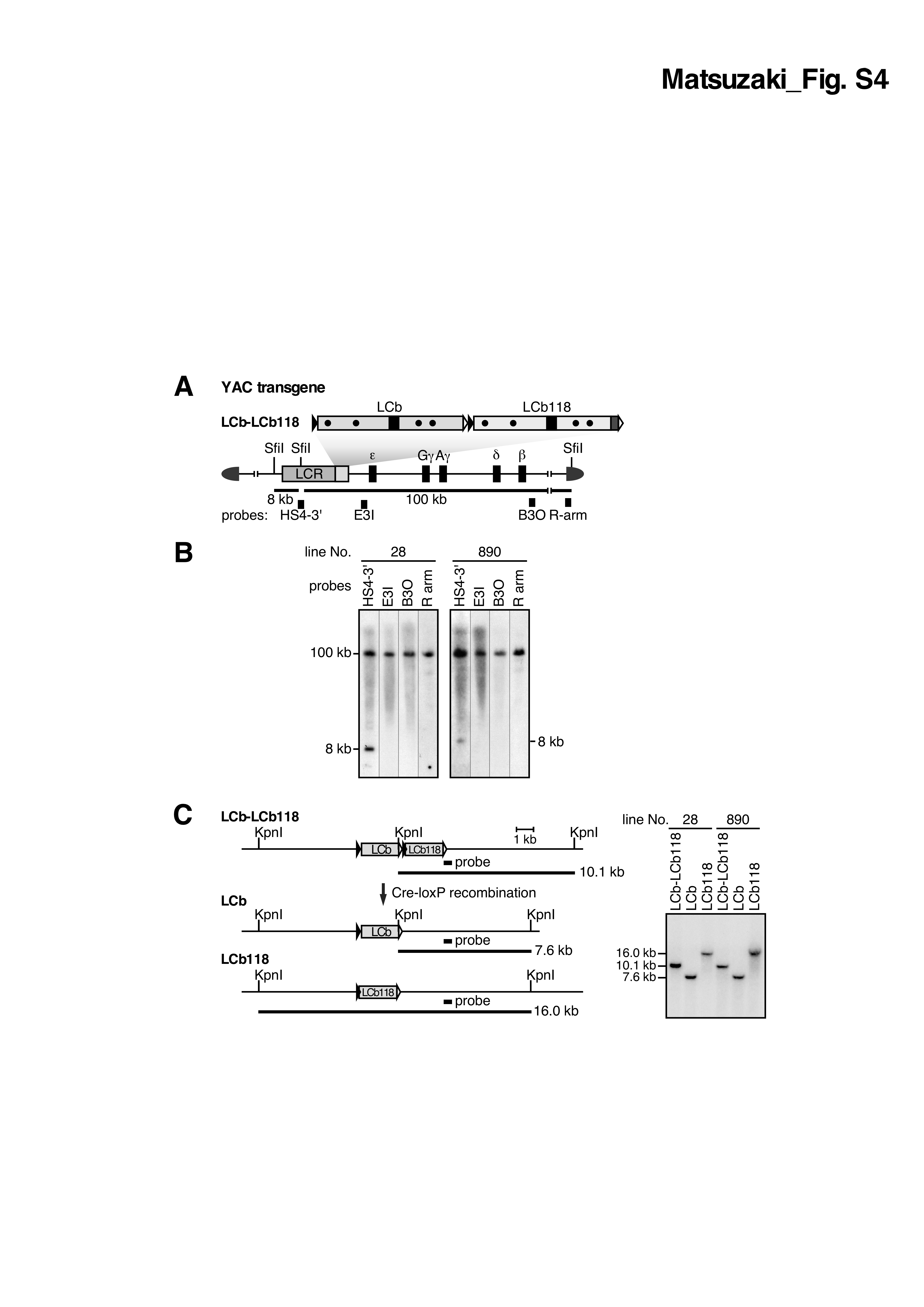

Supplement: Supplementary file 4 — Additional file 4: Figure S4. Generation and structural analysis of YAC-TgM carrying the LCb and LCb118 fragments. (A) Structure of the 150-kb human β-globin locus YAC. The LCR and β-like globin genes are denoted as gray and filled boxes, respectively. The enlarged map shows tandemly arrayed LCb and LCb118 fragments, inserted 3′ to the LCR for employing co-placement strategy. The positions of loxP5171 and loxP2272 are indicated as solid and open triangles, respectively. The expected SfiI restriction enzyme fragments (thick lines) and probes (filled rectangles) used in (B) are shown. (B) Long range structural analysis of the LCb-LCb118 YAC transgene. DNA from thymus cells was digested with SfiI in agarose plugs and separated by pulsed-field gel electrophoresis, and Southern blots were hybridized separately to probes. (C) In vivo Cre-loxP recombination to derive LCb or LCb118 TgM. Tail DNA from parental and daughter YAC-TgM sublines was digested with KpnI and analyzed by Southern blotting using the probe. [file 13072_2019_326_MOESM4_ESM.tif]

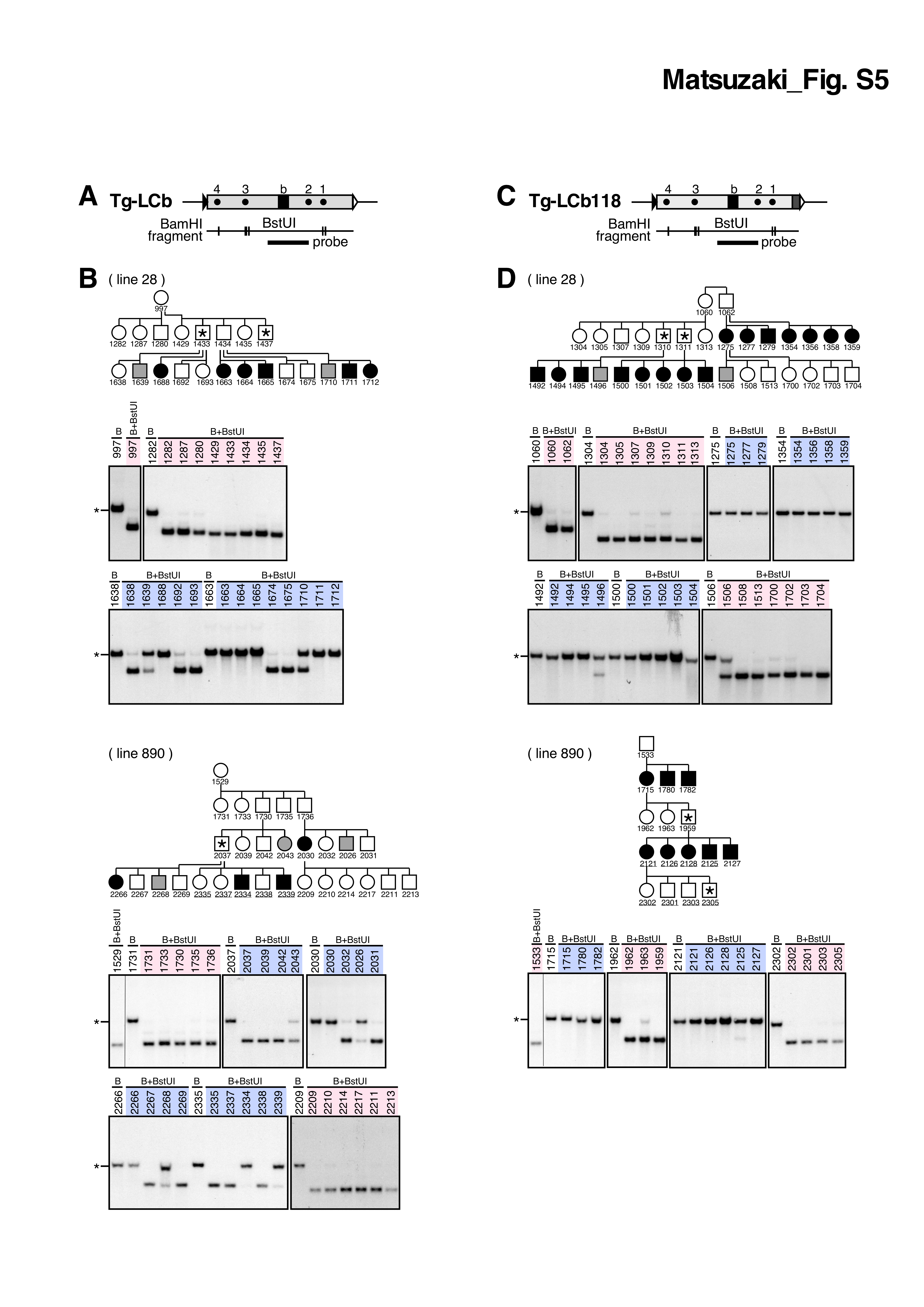

Supplement: Supplementary file 5 — Additional file 5: Figure S5. DNA methylation status of the LCb and LCb118 fragments in somatic cells of YAC-TgM. (A and C) Partial restriction enzyme maps of the β-globin YAC transgenes with the inserted LCb (A) or LCb118 (C) fragments. Methylation-sensitive BstUI sites in BamHI fragments are displayed as vertical lines beneath each map. (B and D) DNA methylation status of the LCb (B) or LCb118 (D) fragments in tail somatic cells of the YAC-TgM. Tail genomic DNA was digested with BamHI alone (B) or BamHI + BstUI (B + BstUI) and the Southern blots were hybridized with the probe shown in the maps (A and C). Asterisks indicate the positions of parental or methylated, undigested fragments. ID numbers of individuals inheriting the transgene maternally and paternally are highlighted in pink and blue colors, respectively. In the pedigree, male and female individuals are represented as rectangles and circles, respectively. Filled, gray, or open symbols indicate hyper-, partially-, or hypo-methylated status of LCb or LCb118 fragment in each TgM, which was independently determined by visual examination of the Southern blot results by three individuals. Tail DNA from underlined animals (in the pedigree) was pooled according to the transgene’s parental origin and analyzed by bisulfite sequencing in Fig. 5b, c. Testis samples in Additional file 6: Fig. S6 were obtained from male individuals marked by stars. [file 13072_2019_326_MOESM5_ESM.tif]

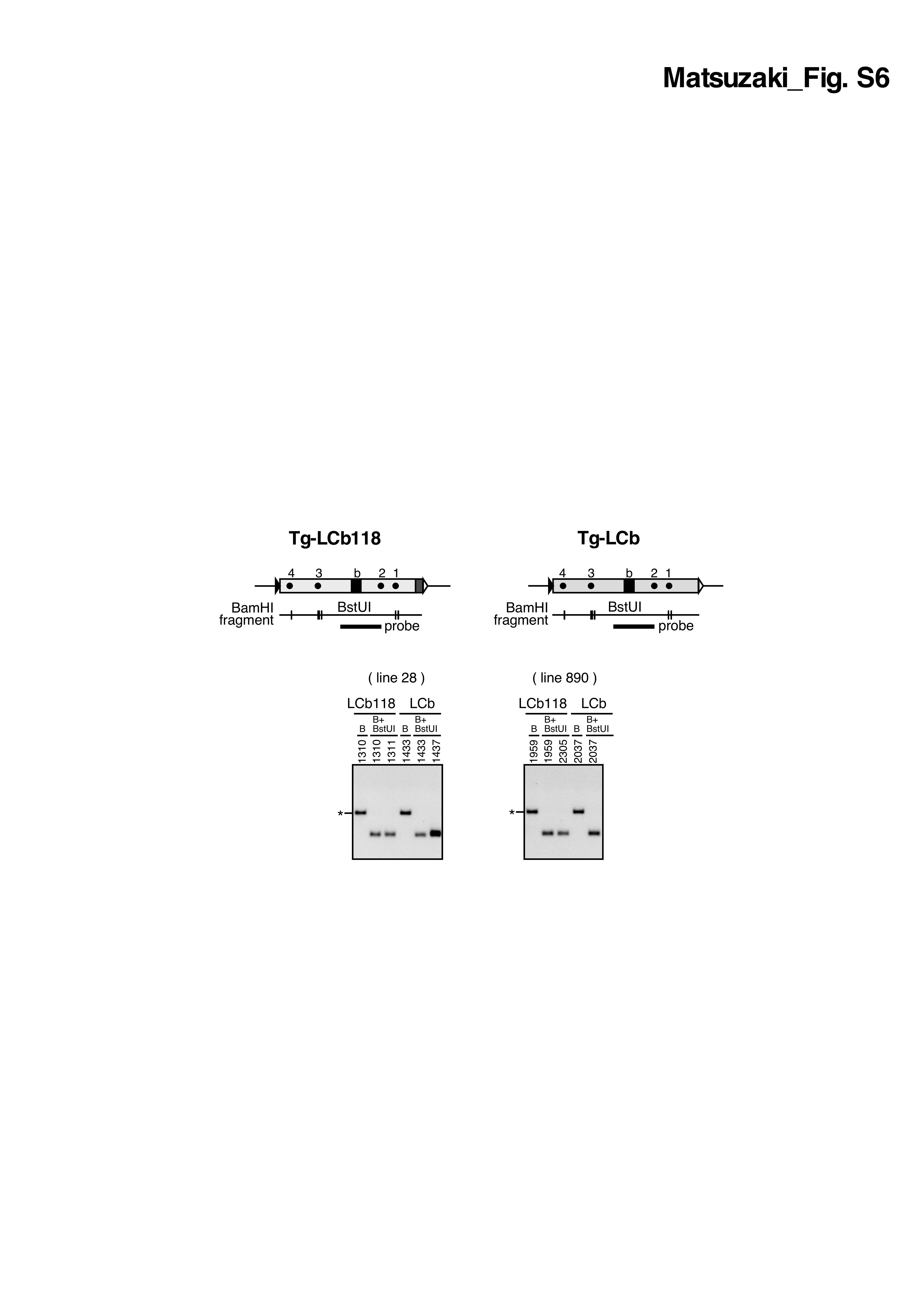

Supplement: Supplementary file 6 — Additional file 6: Figure S6. DNA methylation status of the LCb and LCb118 fragments in testes. Testis genomic DNA from adult male YAC-TgM was analyzed by Southern blotting as described in the legend to Additional file 5: Fig. S5. Sperm samples were obtained from No. 2037 (LCb, line 890) and 1959 (LCb118, line 890) animals, and methylation status of the transgenes were analyzed by bisulfite sequencing in Fig. 5b, c. [file 13072_2019_326_MOESM6_ESM.tif]

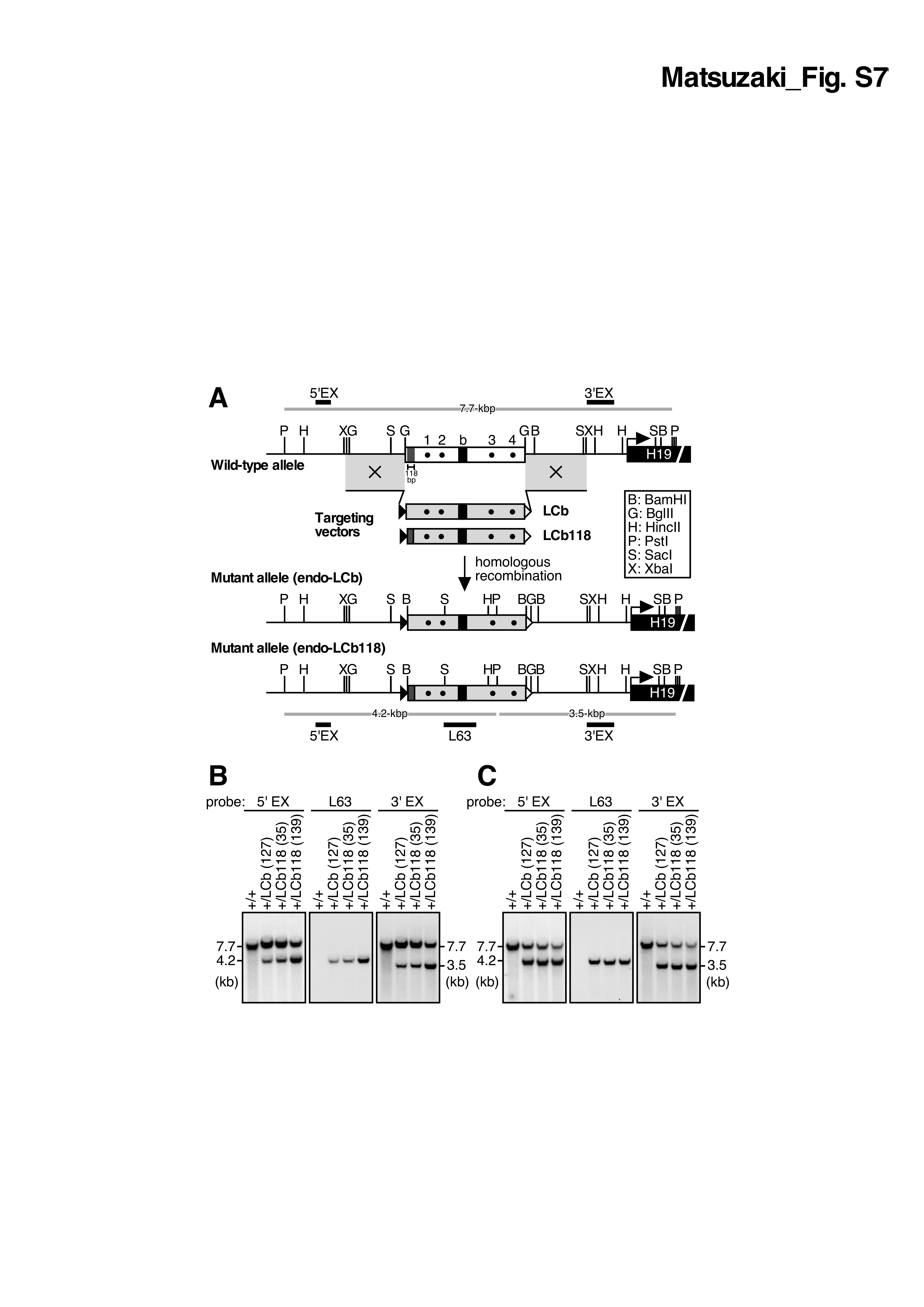

Supplement: Supplementary file 7 — Additional file 7: Figure S7. Generation of LCb/LCb118 knock-in mice. (A) Targeting strategy at the H19 locus. Maps of the wild-type allele, the targeting vectors with the LCb or LCb118 fragment replaced by the H19 ICR, the correctly targeted mutant alleles (endo-LCb and endo-LCb118) are shown from top to bottom. The triangles are the loxP sequences. Probes used for Southern blot analyses in (B) and (C) are shown as filled rectangles. The positions of restriction enzyme sites and expected restriction enzyme fragments with their sizes in each allele are shown nearby. (B, C) Following digestion with PstI and separation on agarose gels, genomic DNA from ES clones (B) and mutant mouse tails (C) on Southern blots were hybridized to one of the three probes, 5′EX, L63, or 3′EX. [file 13072_2019_326_MOESM7_ESM.tif]

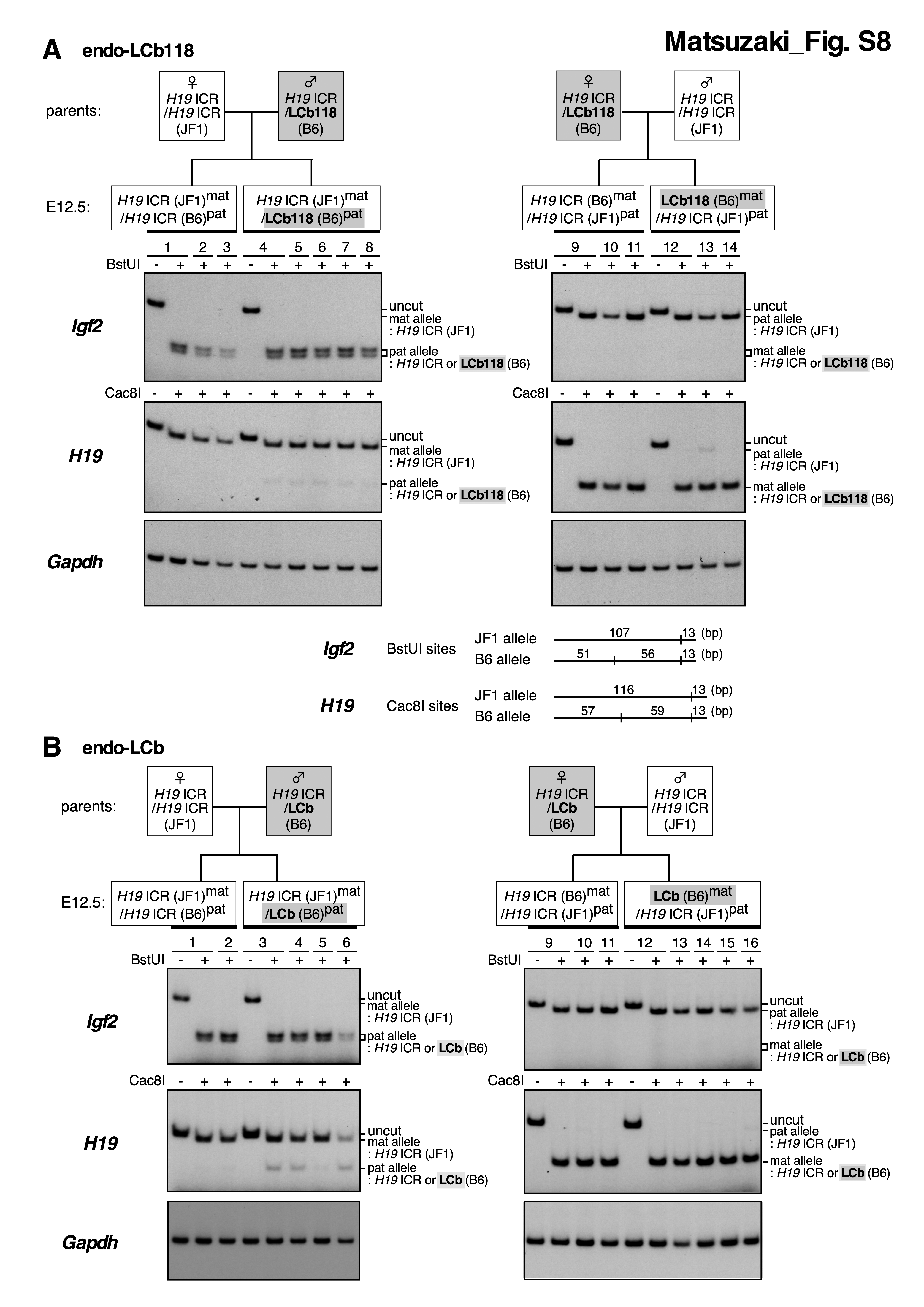

Supplement: Supplementary file 8 — Additional file 8: Figure S8. Monoallelic gene expression pattern is recapitulated in LCb118 knock-in mice. Gene expression analysis of the endo-LCb118 (A) or endo-LCb (B) embryos. In order to distinguish parental origin of the alleles by using SNPs between inbred mouse strains, endo-LCb118 or -LCb hetero-knock-in mice (H19 ICR/LCb118 or LCb; C57BL/6 J [B6] background) were mated with wild-type mice (H19 ICR/H19 ICR; JF1/Msf [JF1]), and offspring was obtained. Total RNA was prepared from livers of E12.5 embryos. Igf2 and H19 gene transcripts were amplified by RT-PCR (within logarithmic amplification range) with α-32P-labeled dCTP, followed by BstUI or Cac8I digestion, respectively. Parental origin of transcripts was discriminated by allele-specific restriction sites. The sites were also introduced into primer sequence so that complete digestion of PCR products can be concomitantly monitored. Gapdh gene transcript was analyzed as control. [file 13072_2019_326_MOESM8_ESM.tif]

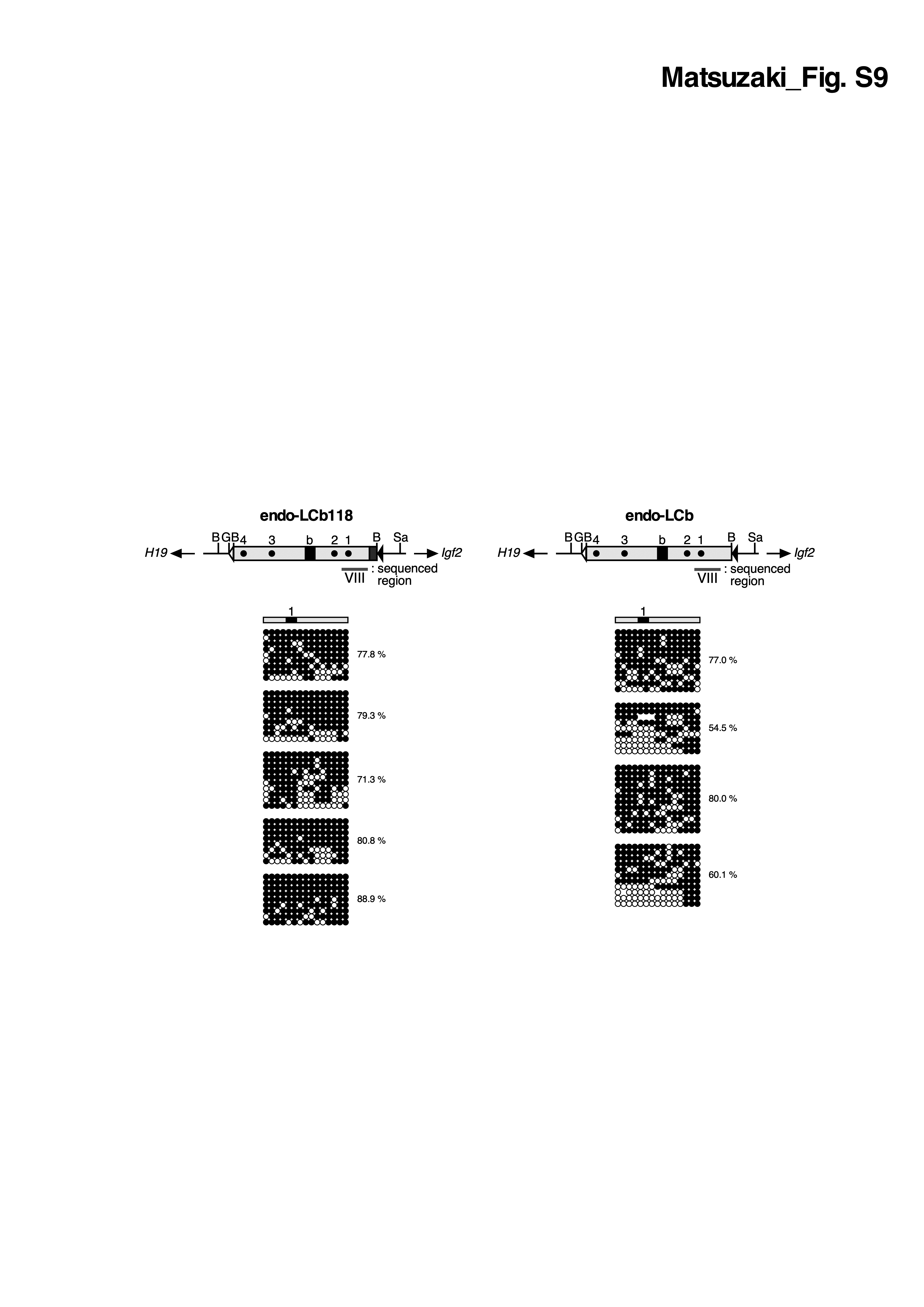

Supplement: Supplementary file 9 — Additional file 9: Figure S9. Genomic imprinting is recapitulated in LCb118 knock-in mice. DNA methylation status of the paternally inherited endo-LCb118 and -LCb sequences. Genomic DNA were extracted from livers of E12.5 embryos, which were analyzed in Additional file 8: Fig. S8, and used for bisulfite sequencing analysis. [file 13072_2019_326_MOESM9_ESM.tif]

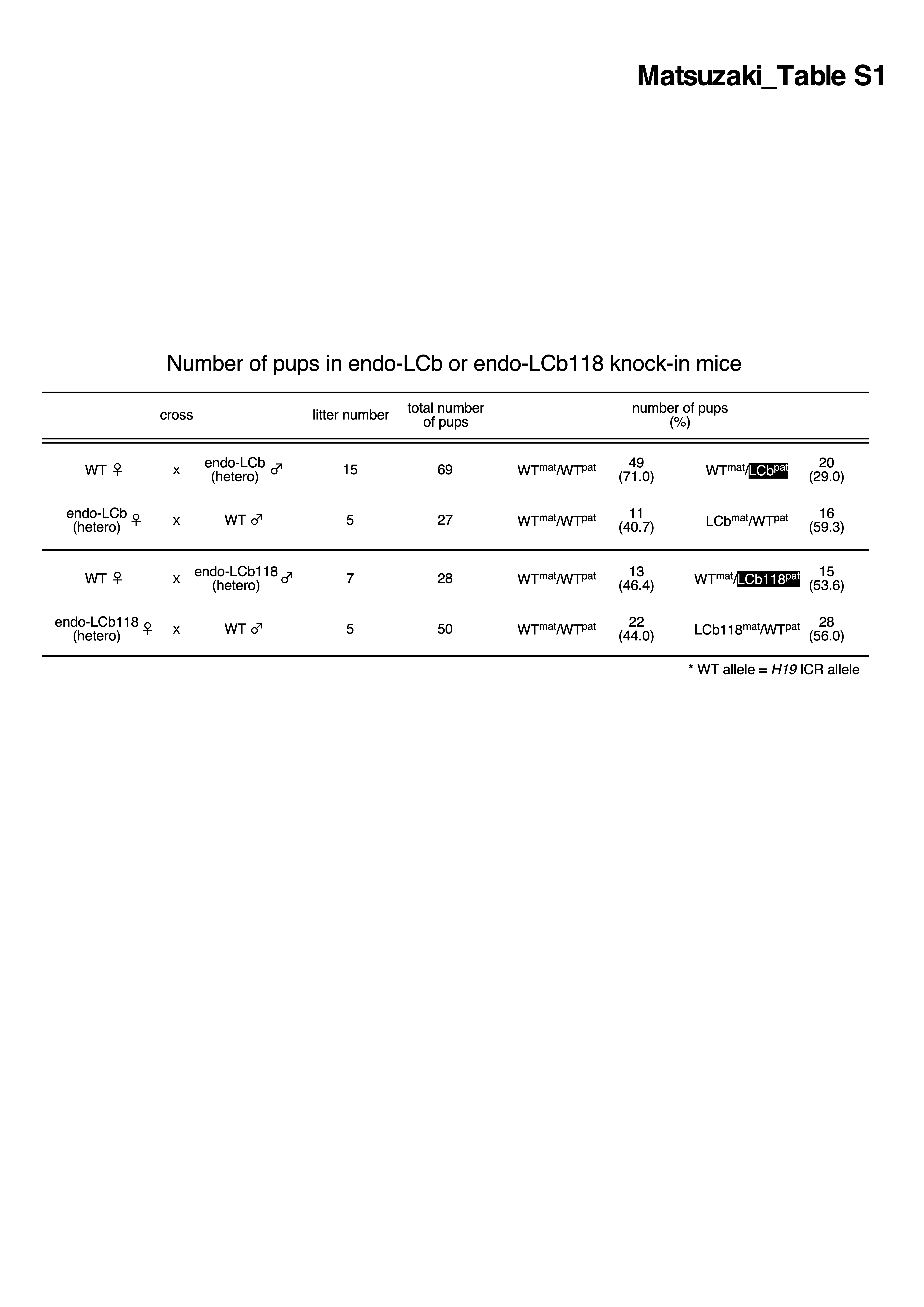

Supplement: Supplementary file 10 — Additional file 10: Table S1. Number of pups in endo-LCb or endo-LCb118 knock-in mice. [file 13072_2019_326_MOESM10_ESM.tif]
